# Regulating the Electrical and Mechanical Properties of TaS_2_ Films via van der Waals and Electrostatic Interaction for High Performance Electromagnetic Interference Shielding

**DOI:** 10.1007/s40820-023-01061-1

**Published:** 2023-04-18

**Authors:** Fukang Deng, Jianhong Wei, Yadong Xu, Zhiqiang Lin, Xi Lu, Yan-Jun Wan, Rong Sun, Ching-Ping Wong, Yougen Hu

**Affiliations:** 1 Shenzhen Institute of Advanced Electronic Materials, Shenzhen Institute of Advanced Technology, Chinese Academy of Sciences, Shenzhen, 518055 People’s Republic of China; 2 Shenzhen Geim Graphene Center, Institute of Materials Research, Shenzhen International Graduate School, Tsinghua University, Shenzhen, 518055 People’s Republic of China; 3 School of Materials Science and Engineering, Georgia Institute of Technology, Atlanta, GA 30332 USA

**Keywords:** 2D transition metal dichalcogenides, 2H-TaS_2_, Flexibility, Electromagnetic interference shielding

## Abstract

Low-dimensional transition metal dichalcogenides (TMDs) have unique electronic structure, vibration modes, and physicochemical properties, making them suitable for fundamental studies and cutting-edge applications such as silicon electronics, optoelectronics, and bioelectronics. However, the brittleness, low toughness, and poor mechanical and electrical stabilities of TMD-based films limit their application. Herein, a TaS_2_ freestanding film with ultralow void ratio of 6.01% is restacked under the effect of bond-free van der Waals (vdW) interactions within the staggered 2H-TaS_2_ nanosheets. The restacked films demonstrated an exceptionally high electrical conductivity of 2,666 S cm^−1^, electromagnetic interference shielding effectiveness (EMI SE) of 41.8 dB, and absolute EMI SE (SSE/t) of 27,859 dB cm^2^ g^−1^, which is the highest value reported for TMD-based materials. The bond-free vdW interactions between the adjacent 2H-TaS_2_ nanosheets provide a natural interfacial strain relaxation, achieving excellent flexibility without rupture after 1,000 bends. In addition, the TaS_2_ nanosheets are further combined with the polymer fibers of bacterial cellulose and aramid nanofibers via electrostatic interactions to significantly enhance the tensile strength and flexibility of the films while maintaining their high electrical conductivity and EMI SE.This work provides promising alternatives for conventional materials used in EMI shielding and nanodevices.

**Supplementary Information:**

The online version contains supplementary material available at 10.1007/s40820-023-01061-1.

## Highlights

- A flexible freestanding TaS_2_ film (thickness = 3.1 μm) exhibits an ultralow void ratio of 6.01%, an ultra-high electrical conductivity of 2,666 S cm^−1^, an electromagnetic interference shielding effectiveness (EMI SE) of 41.8 dB, an absolute EMI SE (SSE/t) of 27,859 dB cm^2^ g^−1^, and excellent flexibility withstand 1,000 bends without rupture.
- The TaS_2_ composite films exhibit excellent EMI shielding properties and higher tensile strength with better mechanical flexibility, making them suitable for EMI shielding practical applications.

**Supplementary Information:**

The online version contains supplementary material available at 10.1007/s40820-023-01061-1.

## Graphical abstract

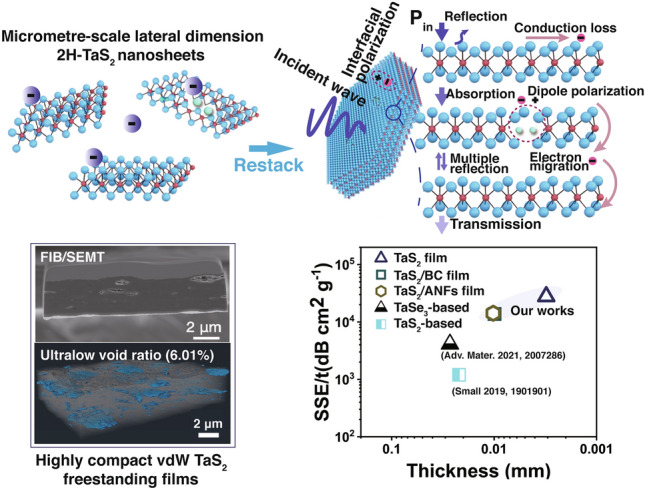

## Introduction

High performance electromagnetic interference (EMI) shielding materials with lightweight, ultrathin thickness, and mechanical flexibility have been an important research field because the development of high-speed communication technology and new wearable electronic devices causes considerable EMI harmful effects on the equipment and human health [1–6]. Two-dimensional (2D) materials are ideal alternatives to traditional metal-based EMI shielding materials, offering both low density and high electrical conductivity [7–14].

Transition metal dichalcogenides (TMDs) are a class of layered 2D materials composed of transition metals and chalcogen elements that interact through van der Waals (vdW) forces [15–17]. Different TMDs exhibit different electrical properties such as semiconducting (2H-MoS_2_, 1T-TaS_2_), semi-metallic (WTe_2_, 1T-TiSe_2_) and metallic (1T′-MoTe_2_, 2H-NbS_2_, 1T-MoS_2_, 2H-TaS_2_) properties [18]. TMDs exhibit different crystal structures, layer numbers, stacking sequences, defect control, and unique 2D morphologies, providing them with excellent physical, chemical, electronic, and optical properties, which enable their use in the fields of electrochemistry [19–21], sensors [22], supercapacitors [23], superconductivity [24–27], thermoelectric [28, 29], electromagnetic wave absorber [30–33] and sieving [34, 35].

Tantalum disulfide (TaS_2_) is one of the popular TMDs materials, in which 2H-TaS_2_ exhibits metallic behavior involving a charge density wave phase transition and superconductivity [24, 36, 37]. Because of its unique electrical properties, it is an ideal material for exploring the effect of electrical conductivity on the EMI shielding performance. High-quality 2D materials must be exfoliated to achieve their full potential. 2H-TaS_2_ can be exfoliated using electrochemical [38, 39], n-butyllithium intercalations [24, 40], mechanical grindings [41], high-boiling-point solvent-assisted ultrasonic methods [37, 42]. However, these methods have several disadvantages such as low efficiency, poor repeatability, and extreme sensitivity to the environment, which makes it difficult to obtain batches of high-quality TaS_2_ nanosheets for freestanding film preparation. Bulk TaS_2_ crystals are rigid and brittle [37, 43], hindering the preparation of flexible TaS_2_ based films. Additionally, 2H-TaS_2_ flakes have poor electron transport along the stacking direction with an out-of-plane and in-plane electrical conductivities of approximately 0.125 and 33,300 S cm^−1^, respectively [44]. Therefore, the development of highly electrically conductive, flexible, and strong restacked TaS_2_ films remains a huge challenge. Despite considerable pioneering efforts devoted to improving both mechanical properties and electrical conductivity of TaS_2_-based films, the tensile strength is often about 10 MPa [37, 45], and the electrical conductivity is only 1173.8 S cm^−1^ [37].

Herein, the metallic 2H-TaS_2_ films with superior mechanical properties, conductivity, and EMI shielding properties were introduced. The 2H-TaS_2_ nanosheets with micrometre-scale lateral dimensions were prepared using a highly feasible intercalation strategy in a highly concentrated LiOH aqueous solution under mild conditions. A flexible freestanding TaS_2_ film with ultrathin thickness of 3.1 μm was successfully restacked through van der Waals interactions, and the film demonstrated an ultra-high electrical conductivity of 2666 S cm^−1^, an EMI SE of 41.8 dB, an absolute EMI SE (SSE/t) of 27,859 dB cm^2^ g^−1^, a high tensile strength of 23.3 ± 4.8 MPa, and excellent flexibility withstand 1000 bends without rupture. In addition, TaS_2_/fiber composite films were also fabricated to further improve the flexibility and strength of the nanosheets while maintaining a high EMI SE. The composite film can be readily folded into a complex shape and unfolded without structural disintegration while effectively shielding against the practical application of 2.4 GHz Bluetooth.

## Experimental Section

### Materials

Bulk tantalum disulfide (2H-TaS_2_, 99.99%) powder was received from Nanjing NXNANO Tech. Co., Ltd. Dimethyl sulfoxide (DMSO, 99.7%), anhydrous lithium hydroxide (99.99% metals basis) was received from Shanghai Aladdin Biochemical Tech. Co., Ltd. Hydrochloric acid (HCl, 36% ~ 38%) was purchased from DONGJIANG Reagent. Poly-p-phenylene terephthamide (PPTA) fibers were obtained from Dupont. Bacterial cellulose dispersion (1 wt%) was obtained from FEYNMAN NANO. Deionized water (DI water, resistivity > 18.2 ${\text{M}}\Omega \cdot {\text{cm}}$) was collected from a Milli-Q Direct-Q 8UV system. All chemicals were used as received without any further purification.

### Exfoliation of 2H-TaS_2_ Nanosheets

2H-TaS_2_ nanosheets were exfoliated by alkaline ion intercalation method. Tantalum disulfide (2H-TaS_2_) powder (0.1 g) was mixed with lithium hydroxide solution (1 mL, 2 M) in centrifugal tube for 9 h at room temperature. The final mixture was then washed 3 times using DI water by centrifugation at 12,000 rpm for 15 min until the pH of supernatant is about 7. Subsequently, the resulting swelled sediment was diluted with 80 mL DI water and sonicated for 1 h in an ice bath. Finally, the TaS_2_ nanosheets aqueous dispersion (~ 1.25 mg mL^−1^) was obtained without centrifugation.

### Van der Waals TaS_2_ Freestanding Films

To prepare pristine TaS_2_ freestanding films, the fresh 2H-TaS_2_ nanosheets aqueous dispersions (~ 1.25 mg mL^−1^) was subjected to vacuum filtration using polycarbonate micro-porous membrane (Whatman) as substrates, followed by drying at 50 °C for 10 h. The freestanding TaS_2_ ultrathin films with thickness of about 3.1 μm were finally obtained by peeling off from the substrates.

### Synthesis of TaS_2_ Composite Films

The bacterial cellulose (BC) dispersion (1 wt%) was added to HCl solution (0.01 M) protonation for 30 min, and ultrasonication for 1 min before use. Then the freshly synthesized TaS_2_ nanosheets dispersion was mixed with BC/HCl dispersion. The resulting suspension was then vacuum filtered after hand shaking 1 min using polyethersulfone membranes as substrates. Subsequently, the pre-preparation TaS_2_/BC composite film was washed with 10 mL of DI water by continuing vacuum filtration and dried at 50 °C for 10 h to form a final TaS_2_/BC composite film. Based on the addition of BC, the following five types of TaS_2_/BC composite films with various mass ratios were prepared: TaS_2_/BC (10:1), TaS_2_/BC (10:2), TaS_2_/BC (10:3), TaS_2_/BC (10:4), and TaS_2_/BC (10:5).

The ANFs fibers were fabricated by proton donor-assisted deprotonation [46]. Five types of TaS_2_/ANFs composite films with the same weight ratios as TaS_2_/BC composite films above were prepared: TaS_2_/ANFs (10:1), TaS_2_/ANFs (10:2), TaS_2_/ANFs (10:3), TaS_2_/ANFs (10:4), and TaS_2_/ANFs (10:5).

### Materials Characterizations

The structures of bulk 2H-TaS_2_ and the films were characterized by X-ray diffraction (XRD, Bruker, D8 Advance X using Cu K $\alpha$ radiation) and RAMAN spectrometer (LabRAM HR Evolution, HORIBA). The morphology and thickness of as-synthesized films were characterized by scanning electron microscopy (SEM, Thermo Scientific, Apero 2 S HiVac). The elemental morphology and compositions of TaS_2_ and TaS_2_ composite films were detected by X-ray photoelectron spectroscopy (XPS, ESCALAB 250XI+). The elemental composition was characterized by ICP–OES (Agilent 7700) and NMR (Bruker Avance III 500 MHz WB). High-angle annular dark field scanning transmission electron microscopy (HAADF–STEM, FEI Talos F200X G2) was used to characterize the TaS_2_ nanosheets. The thickness of the exfoliated nanosheets was measured using atomic force microscopy (AFM, Bruker, Dimension ICON). The zeta potential of dispersions was measured on Malvern Zetasizer Nano ZS90. Hydrophilicity of the films was analyzed at 298 K using a contact angle analyzer (OCA20, DataPhysics). The mechanical properties of films were investigated by using dynamic mechanical analysis (DMA850, TA).

To detect the 3D reconstruction microstructure of the TaS_2_ freestanding films and TaS_2_ composite films, a layer of tungsten was deposited on the upper surface of the films, then cut by a focused ion beam (FIB) to provide cross-sections using a FEI Helios NanoLab 600i (using an acceleration voltage of 30 kV and a current of 2.4 nA). Due to the difference in height between the voids and cross-section, and the difference in atomic number between the polymer and TaS_2_, the contrast is different. The serial backscattering electron section images of TaS_2_ freestanding films and TaS_2_ composite films were obtained by FIB/SEM tomography (FIB/SEMT) with a constant separation of 30 nm (using an acceleration voltage of 5 kV and a current of 0.8 nA). Finally, the software (Thermo Scientific Auto Slice&View 4 and Avizo) was used to reconstruct the corresponding three-dimensional (3D) microstructure and automatically calculate all data.

### Electrical Conductivity Measurement

The electrical conductivity corresponding to the pressure of TaS_2_ powder was measured using a powder resistivity system (PRCD2000, IEST Co., Ltd). Electrical conductivity of all TaS_2_ films were measured using a non-contact resistivity tester (EC−80P, NAPSON CORPORATION). The electrical conductivity of all TaS_2_ films were calculated by the Eq. (1):

1 $$\sigma =1/({R}_{s}t)$$

where $\sigma$ is the electrical conductivity [S cm^−1^], ${R}_{s}$ is the sheet resistance [Ω sq^−1^] and $t$ is the thickness of samples [cm]. Thickness measurements were performed by using a highly accurate length gauge ($\pm 0.01$ μm, VL-50-B, Mitutoyo, Japan) and counter checked by the SEM technique. The density of pure TaS_2_ and composite films was calculated from experimental measurements of the volume and mass of the samples.

### Electromagnetic Interference Shielding Characterization

EMI measurements of pristine as well as composite films were carried out in a rectangular waveguide (HD-100WCAS, HD Microwave) using PNA network analyzer (PNA-N5227B, Keysight, USA) in X-band frequency range (8.2–12.4 GHz).

The reflection (*R*), absorption (*A*), and transmission (*T*) coefficients were calculated by scattering parameters (*S*_11_, *S*_22_, *S*_12_, and *S*_21_ obtained from the PNA network analyzer) as:

2 $$R={\left|{S}_{11}\right|}^{2}$$

3 $$T={\left|{S}_{21}\right|}^{2}$$

4 $$A=1-T-R$$

Furthermore, the total EMI SE (SE_*T*_), microwave reflection (SE_*R*_), and microwave absorption (SE_*A*_) can be calculated from *R* and *T* coefficients as:

5 $${SE}_{T}=-10\mathrm{log}T$$

6 $${SE}_{R}=-10\mathrm{log}(1-R)$$

7 $${SE}_{A}=-10\mathrm{log}\left(\frac{T}{1-R}\right)= {SE}_{T}-{SE}_{R}-{SE}_{M}$$

The absolute effectiveness (SSE/t) were calculated by the Eq. (8) [7, 47, 48]:

8 $${\text{SSE}}/{\text{t}}\, = \,{\text{EMI SE}}/{\text{density}}/{\text{t}}\, = \,{\text{dB cm}}^{{2}} {\text{g}}^{{ - {1}}}$$

## Results and Discussion

### Characterization of 2H-TaS_2_ Nanosheets

**Fig. 1:**
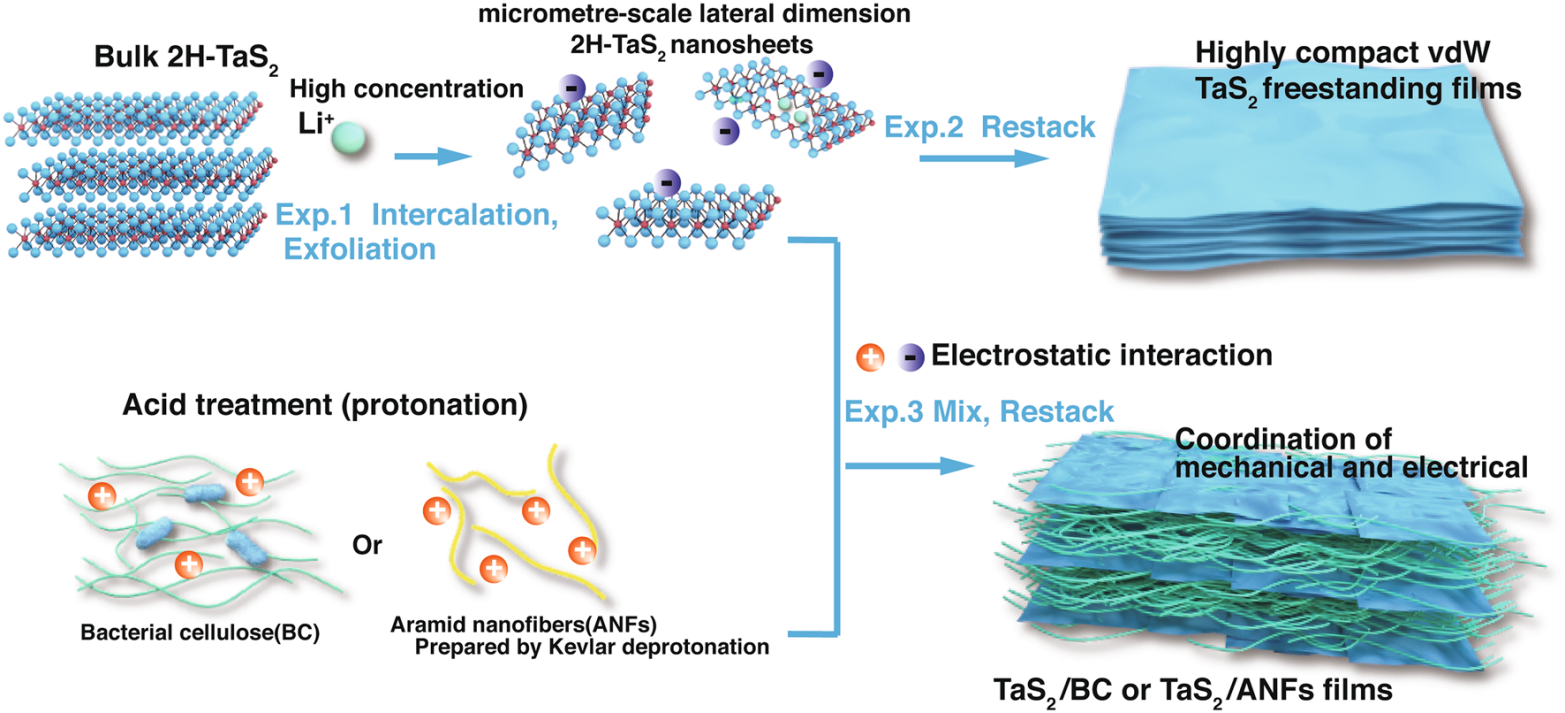
Schematic illustrating the preparation processes of TaS_2_ freestanding films, TaS_2_/bacterial cellulose (BC) or aramid nanofibers (ANFs) nanocomposite films

**Fig. 2:**
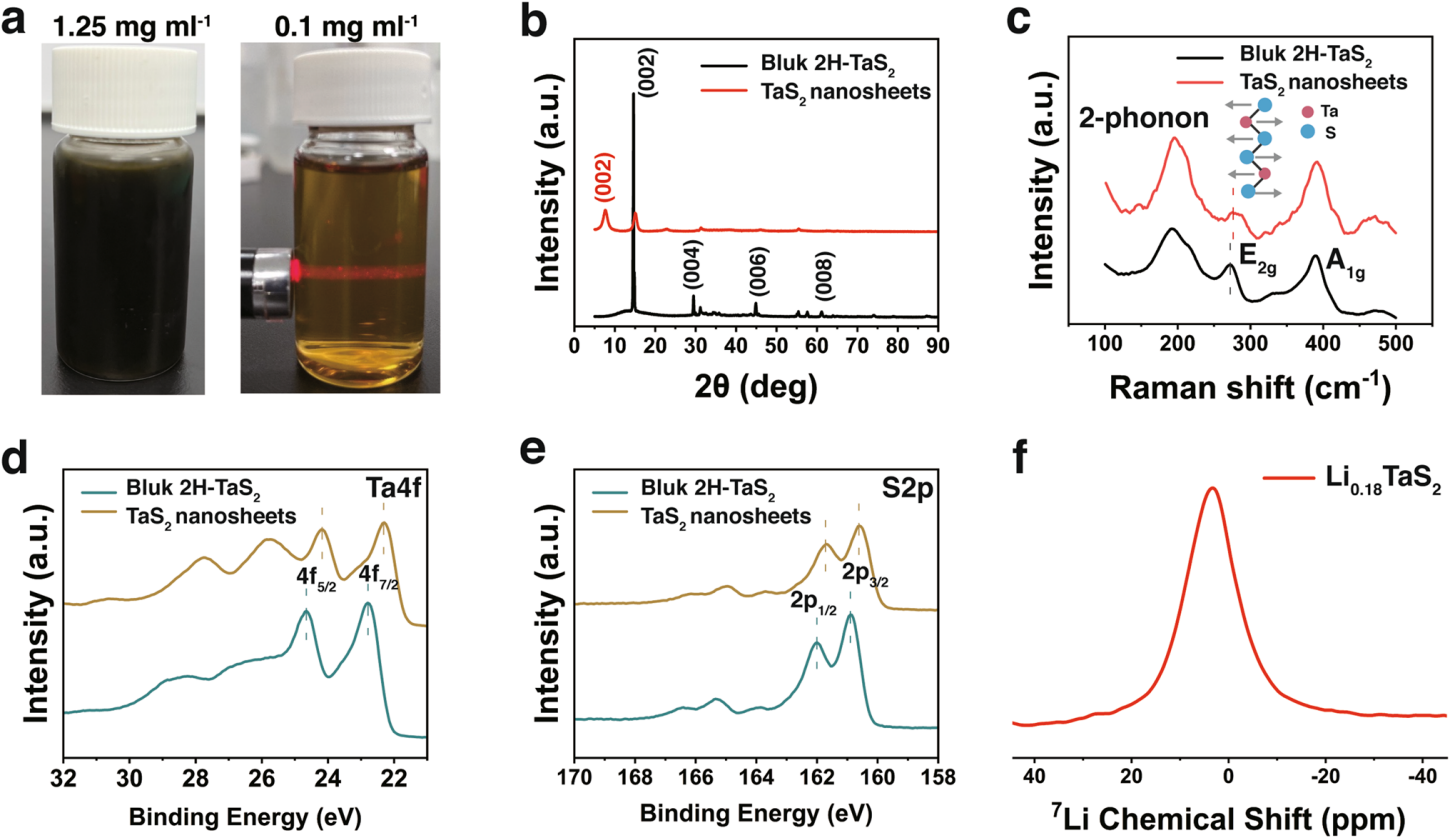
**a** Exfoilated 2H-TaS_2_ nanosheets aqueous dispersions. **b** XRD spectra of 2H-TaS_2_ crystals and lyophilized 2H-TaS_2_ nanosheets. **c** Raman spectroscopy analysis of the exfoliated nanosheets and the bulk crystal for comparison. XPS high-resolution spectra of **d** Ta 4*f* and **e** S 2*p* of 2H-TaS_2_ crystals and lyophilized 2H-TaS_2_ nanosheets. **f** ^7^Li SP MAS NMR spectra of 2H-TaS_2_ nanosheets

The 2H-TaS_2_ nanosheets were produced by Li-ion intercalation, which involves immersing 2H-TaS_2_ crystals in a highly concentrated lithium hydroxide solution at room temperature followed by a mild sonication and exfoliation processes (Fig. 1 for more details, refer to the Experimental Section). Unlike conventional organic-solvent and n-butyllithium intercalations, this method is scalable and safe, and it does not involve time-consuming or complex processes. Using Li ions in the intercalation process involves electron transfer from the *s* orbitals of the Li ions to the *d* orbitals of the transition metal atoms [38]. Therefore, the high concentration of the intercalated Li leads to the injection of a massive number of electrons into the TaS_2_ crystal, resulting in the retention of the intrinsic metallic 2H crystalline phase in TaS_2_ [17, 40, 49–54] The concentrated solution of the exfoliated TaS_2_ nanosheets appears black, whereas the diluted TaS_2_ aqueous dispersion (~ 0.1 mg mL^−1^), in which the Tyndall effect is observed, appears yellow (Fig. 2a), indicating the formation of relatively thin nanosheets. The formation of stable dispersions is attributed to the electrostatic repulsion between the nanosheets, which have a high negative charge (Fig. S1).

The metallic 2H phase of the lyophilized TaS_2_ nanosheets was confirmed by XRD measurements (Fig. 2b) according to the PDF #80–0685 indexes [24, 55]. The (002) sharp peak (full width at half-maximum (FWHM) = 0.21°) indicated an excellent crystallinity of the bulk 2H-TaS_2_. The broadening of the XRD peaks may be related to the extent of the crystal domain (a broader peak reflects a smaller crystal domain) [56]. The (002) peak of the lyophilized TaS_2_ nanosheets (FWHM = 1.50°) was broader than that of the 2H-TaS_2_ crystals, which indicated the successful exfoliation of the sample and the presence of vdW restacking effects between the TaS_2_ nanosheets. Raman spectroscopy (Fig. 2c) confirmed the crystallinity retention of the exfoliated 2H-TaS_2_ [24, 40, 56]. Two peaks of TaS_2_ nanosheets were observed at approximately 392.0 and 275.8 cm^–1^, which correspond to the out-of-plane vibration (A_1g_) and the in-plane vibration (E_2g_) modes, respectively, of 2H-TaS_2_ at room temperature, and a broad second-order peak, which is attributed to a two-phonon scattering process, was observed at approximately 196.2 cm^−1^. The E_2g_ peak of the lyophilized TaS_2_ nanosheets was slightly blue shifted to 3.8 cm^−1^ compared to that of the bulk 2H-TaS_2_, indicating the effective exfoliation of TaS_2_ nanosheets [55]. In addition, the integral E_2g_/A_1g_ ratio of the TaS_2_ nanosheets is 0.107, which is very close to that of the monolayer [37].

The XPS further confirmed the presence of the 2H phase in the bulk TaS_2_ powder. As shown in Fig. 2d, the doublets at 22.8 and 24.7 eV are assigned to Ta^4+^ 4*f*_7/2_ and Ta^4+^ 4*f*_5/2_, respectively, of 2H-TaS_2_ [19, 57]. The peaks at 160.9 eV (2*p*^3/2^) and 162.0 eV (2*p*^1/2^) (Fig. 2e), which are the signature peaks of S^2+^ in 2H-TaS_2_, were observed [57]. As shown in Fig. 2d–e, the Ta 4*f* and S 2*p* peaks shift toward a lower bonding energy, indicating that the 2H-TaS_2_ nanosheets obtained electrons form the s orbitals of the Li ions during the intercalation process [39, 55]. This suggests the excellent intercalation and exfoliation of the nanosheets.

The chemical composition of the 2H-TaS_2_ nanosheets (Table S1) was further characterized using an inductively coupled plasma optical emission spectrometer (ICP–OES), and the chemical compositions of the 2H-TaS_2_ nanosheets is found to be Li_0.18_TaS_2_. The presence of Li ions was also confirmed by nuclear magnetic resonance (Fig. 2f). The single sharp ^7^Li signal at approximately 3.49 ppm indicates that Li ions were all in the same chemical environment.

**Fig. 3:**
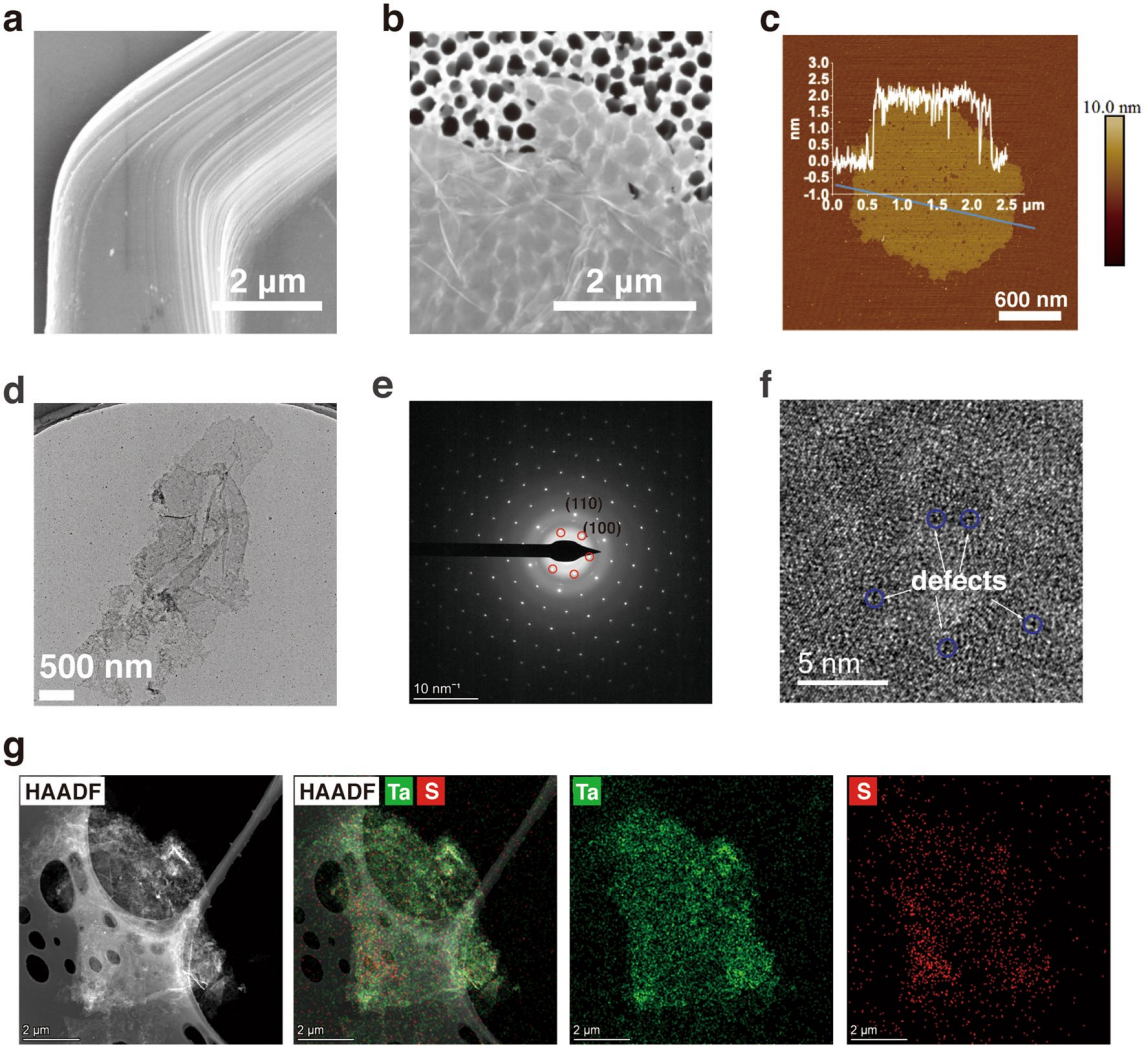
SEM image of the **a** 2H-TaS_2_ crystals and **b** 2H-TaS_2_ nanosheets. **c** A typical AFM image of 2H-TaS_2_ nanosheets, showing thickness of ~ 1.75 nm. **d** TEM image, **e** selected-area electron diffraction (SAED) image, **f** high-resolution TEM image of 2H-TaS_2_ nanosheets. **g** HAADF-STEM and EDS images of 2H-TaS_2_ nanosheets

The morphology of 2H-TaS_2_ was characterized using SEM (Figs. 3a and S2a–d). The bulk 2H-TaS_2_ is well crystallized with a large layered lateral structure, and the edge of each individual crystal is clearly visible. The composition of the material was investigated using energy-dispersive X-ray spectroscopy (EDS) analysis (Fig. S2e–f), which verified the homogeneous distribution of Ta and S elements throughout the material. The morphology of the 2H-TaS_2_ flakes was characterized using SEM, transmission electron microscopy (TEM), and AFM. Figure 3b shows the SEM image of the exfoliated 2H-TaS_2_ nanosheets, which indicates that they are flexible with large lateral dimensions. SEM statistical analysis (Fig. S3) shows the values of the lateral dimensions of the nanosheets (0.5–11 μm), mainly distributed at values < 5 μm (log-normal distribution peaks at approximately 1.5 μm). Figure 3c shows an AFM image of an individual TaS_2_ nanosheet (thicknesses ≈ 1.75 nm). The figure indicates that few porous defects are observed on the surface of the 2H-TaS_2_ nanosheets. The AFM statistical analysis of the thickness of the 2H-TaS_2_ nanosheets (Fig. S4) shows that the 2H-TaS_2_ nanosheets mainly consist of few layers (the thickness of each TaS_2_ monolayer is generally between 0.4 and 0.9 nm [56]), and TaS_2_ monolayers are also observed in the exfoliated samples.

The low contrast in the TEM image also indicates the thin flake-like nature of the 2H-TaS_2_ nanosheets (Fig. 3d). The hexagonal diffraction spots (Fig. 3e) and the lattice-resolved TEM image (Fig. 3f) indicate the high crystallinity of the 2H-TaS_2_ nanosheets. According to Fig. 3f, few sub-nanopores were also distributed over the substrate of the 2H-TaS_2_ nanosheets, indicating that a high Li-ion intercalation can cause defects. High-angle annular dark field–scanning transmission electron microscope (HAADF–STEM) and EDS images of the 2H-TaS_2_ nanosheets confirm the presence of the Ta and S elements (Fig. 3g).

### Structure Characterization and Mechanical Properties of TaS_2_ Films

**Fig. 4:**
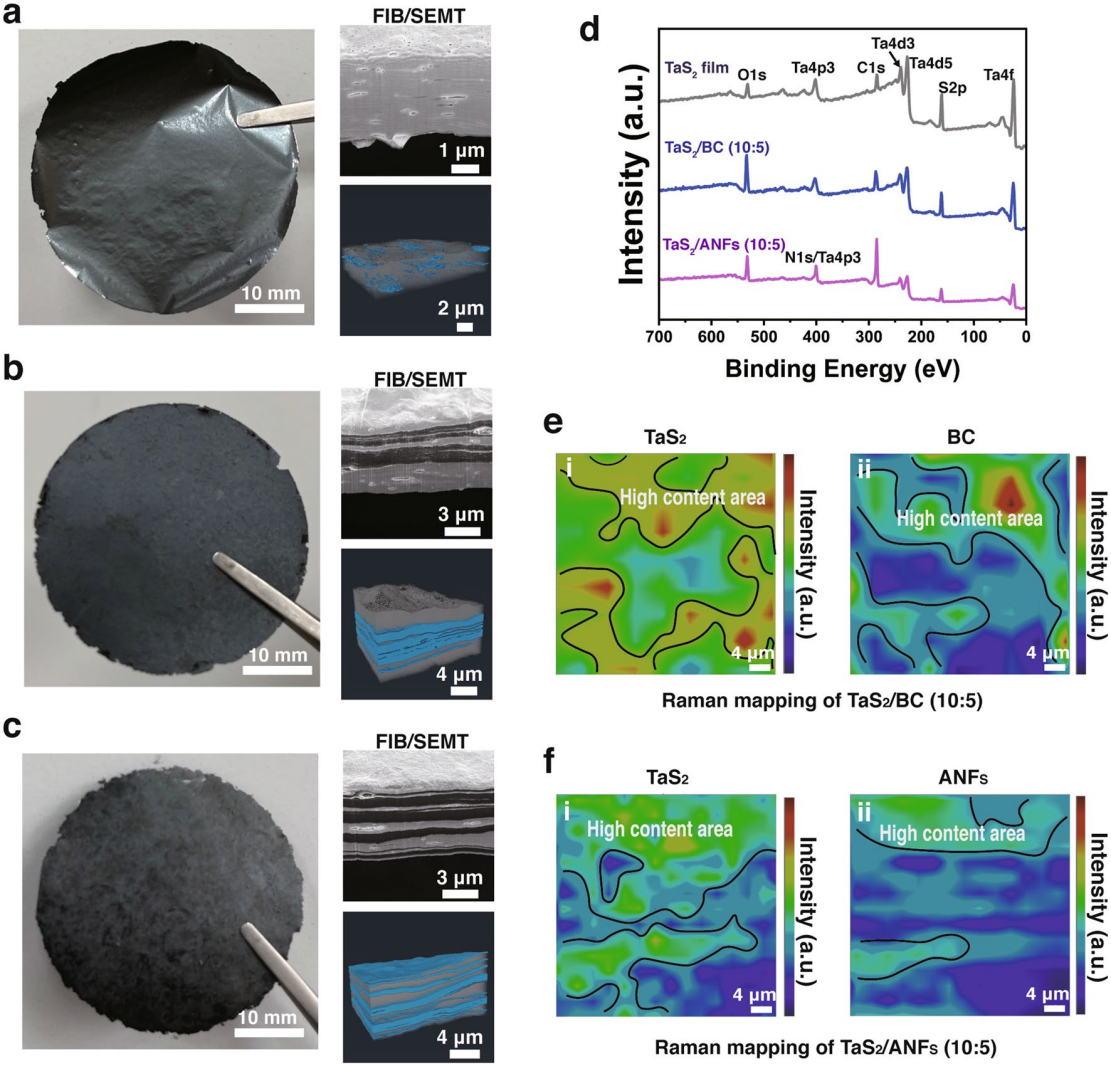
Structure characterizations of TaS_2_ films. Photos, cross-sectional SEM images and 3D reconstruction microstructure based on FIB/SEMT of **a** TaS_2_ freestanding film, **b** TaS_2_ /BC (10:1) composite film, and **c** TaS_2_ /ANFs (10:1) composite film (Gray indicates TaS_2_, Blue indicates voids, BC or ANFs). **d** XPS survey spectra of TaS_2_ freestanding film, TaS_2_ /BC (10:5) composite film, and TaS_2_ /ANFs (10:5) composite film. Raman mappings of **e** TaS_2_/BC (10:5) composite film and **f** TaS_2_/ANFs (10:5) composite film

The TaS_2_ freestanding and composite films were fabricated from their dispersions by vacuum filtration (Fig. 1, for more details, refer to the Experimental Section). In case of the TaS_2_ freestanding films, a perfect TaS_2_ ultrathin film (thickness = 3.1 μm) was prepared (Fig. 4a). Figure S5 shows the morphology and element distribution of a section of the film. The cross-sectional images observed by focused ion beam–SEM (FIB–SEM) revealed that the TaS_2_ film has well-ordered and compact lamellar structure. The mechanical and electrical properties of the restacked nanosheet films are affected by their internal structure [43, 58]. Therefore, the 3D void microstructure of the TaS_2_ films was reconstructed using FIB/SEMT (Fig. S6, Movie S1). Figure S7 shows the volume distribution of the 3D reconstructed voids. The FIB/SEMT results indicate that the ultralow porosity of the TaS_2_ films (6.01%) is significantly lower than that of the MXene films (15.4 ± 0.6%) [58].

Bacterial cellulose (BC) and aramid nanofibers (ANFs) are considered promising matrix materials because of their high stability, good film-forming properties, and high mechanical properties [3, 59–61]. Therefore, we investigated the enhancement effects of BC and ANFs on the mechanical properties of bond-free TaS_2_. Figures 4b, c and S8 show the morphology of the TaS_2_/bacterial cellulose (BC) and TaS_2_/aramid nanofibers (ANFs) composite films. The TaS_2_ freestanding film exhibits a smoother surface than that of the TaS_2_/BC (10:5) composite film and a rougher surface than that of the TaS_2_/ANFs (10:5) composite film. This is attributed to the larger roughness of the pure BC film and the very smaller roughness of the pure ANFs film (Fig. S9). Therefore, the surface roughness of composite film will be between pure fiber film and TaS_2_ film. The real content of BC and ANFs in the TaS_2_ composite films was examined by thermogravimetric analysis (Fig. S10), and the results are presented in Table S2. An acid pretreatment of BC and ANFs allows for the protonation of the fiber, which promotes the aggregation of the TaS_2_ nanosheets around the fiber surface by electrostatic interaction (Fig. S11). This effectively prevents the delamination of the composite films (Fig. S12) due to the huge density difference between the TaS_2_ nanosheets and fibers. In addition, the preparation efficiency of the composite films is greatly accelerated by acid treatment (Movie S2). The cross-sectional SEM and FIB/SEMT images (Figs. 4b, c, S6, and S13) and movies (Movies S3 and S4) of the TaS_2_/BC and TaS_2_/ANFs composite films reveal that both films have alternating multilayer stack structures between TaS_2_ and the fibers. This special structure of the composite films can effectively improve the tensile strength and ensure its high electrical conductivity. As shown in Fig. 4d, the XPS spectra reveal that, based on the increased content of the C and O elements and the occurrence of the N element in the composite films, the BC fibers or ANFs had been successfully introduced into the 2H-TaS_2_ nanosheet layers. The intensity distribution of each component on the surface of TaS_2_/BC (10:5) and TaS_2_/ANFs (10:5) composite films were analyzed by Raman microscope (the size of observation area is 50 μm × 50 μm). As shown in Fig. S14, the shifts at 350–450, 1092, and 1648 cm^−1^ correspond to TaS_2_ [24], C–O of BC [62], and C−N and N−H of ANFs [46], respectively, and the above characteristic peaks are used as signal sources for Raman imaging (Fig. 4e, f). It is observed that TaS_2_ has the local high content area in TaS_2_/BC (10:5) or TaS_2_/ANFs (10:5) composite films (Fig. 4e(i) and f(i)), indicating that TaS_2_ presents non-continuous distribution in the composite. BC and ANFs are also unevenly distributed in the corresponding composite film (Fig. 4e(ii) and f(ii)). This uneven distribution is the reason for the alternating multilayer stack structures between TaS_2_ and the fibers of the composite film (Fig. 4b, c).

**Fig. 5:**
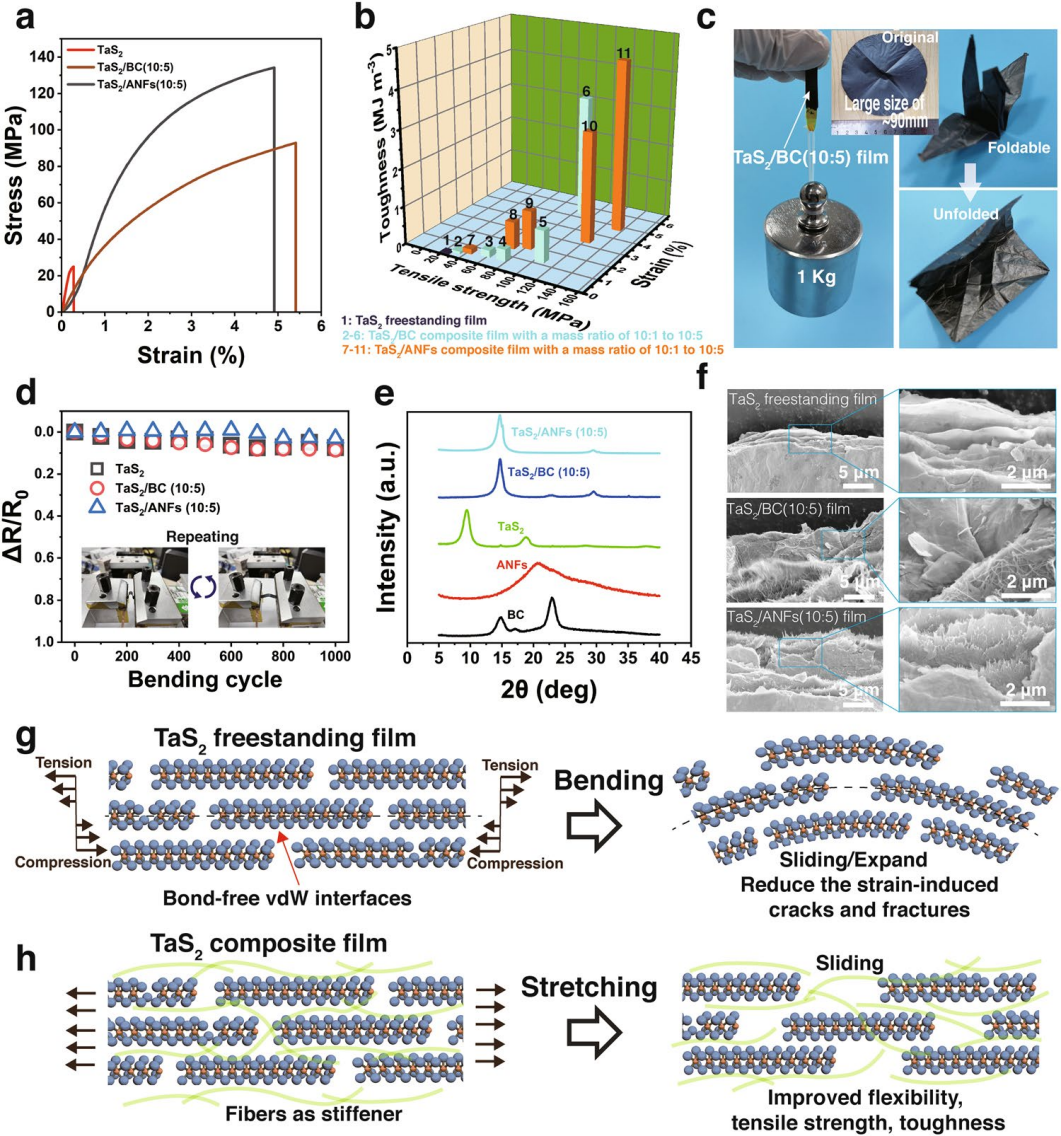
Mechanical properties of TaS_2_ films. **a** Representative tensile stress–strain curves of TaS_2_ freestanding film, TaS_2_/BC (10:5), and TaS_2_/ANFs (10:5) composite films. **b** Comparison of the strain, tensile strength, and toughness of TaS_2_ freestanding film, TaS_2_ /BC, and TaS_2_/ANFs composite films, respectively. **c** Digital images of TaS_2_/BC (10:5) composite films showing their strength and flexibility. **d** Mechanical and electrical stability of TaS_2_ freestanding film, TaS_2_/BC (10:5), and TaS_2_/ANFs (10:5) composite films as a function of the bending cycle. **e** Normalized XRD patterns of BC, ANFs, TaS_2_ freestanding film, TaS_2_/BC (10:5), and TaS_2_/ANFs (10:5) composite films. **f** SEM images of the fracture surfaces of TaS_2_ freestanding film, TaS_2_/BC (10:5), and TaS_2_/ANFs (10:5) films. **g** Schematic diagram of TaS_2_ freestanding film before and after bending. **h** Schematic diagram of TaS_2_ composite films before and after stretching

Figures 5a and S15 show the tensile stress–strain curves of TaS_2_ freestanding film and the TaS_2_/fiber composite films. The tensile strength, Young’s modulus, and toughness of the TaS_2_ freestanding film are 23.3 ± 4.8 MPa, 14.9 ± 6.2 GPa, and 0.033 ± 0.018 MJ m^−3^, respectively (Fig. S16 and Table S3). The tensile strength of the TaS_2_ freestanding film is much higher than that of the previously reported TaS_2_HA_0.371_NMF_0.135_ foil (9.16 MPa) [37], and the PEO/TaS_2_ (0.5 wt%) film (11.27 MPa) [45], which implies that the densified structure and reinforced interlayer interaction between the TaS_2_ nanosheets improved the mechanical properties of the TaS_2_ freestanding film. The TaS_2_/BC (10:5) and TaS_2_/ANFs (10:5) composite films exhibit superior mechanical properties, i.e., their tensile strength is 87.9 ± 8.1 and 134.13 ± 1.4 MPa, respectively, and their toughness is 3.25 ± 0.45 and 4.52 ± 0.07 MJ m^−3^, respectively, which are the highest values reported for TaS_2_ composite films (Fig. 5b and Table S4). Figure 5c demonstrates that the TaS_2_/BC (10:5) composite film can easily withstand a tensile force of 1 kg. The large sized TaS_2_/BC (10:5) composite film (diameter ≈ 90 mm) can be rapidly prepared by our facile method (Fig. 5c, insert picture). The film is smooth, flexible, and can be readily folded into a complex shape and unfolded without structural disintegration. Moreover, the TaS_2_ composite films exhibit a higher tolerance to ultrasound than the TaS_2_ films (Fig. S17), which can be attributed to the better anti-wettability of the composite films and stronger interaction between the TaS_2_ nanosheets and fibers compared to those in case of the pure TaS_2_ films (Fig. S18). Figure 5d indicates that the relative change in the resistance of the TaS_2_ freestanding film, TaS_2_/BC (10:5) composite films, and TaS_2_/ANFs (10:5) composite films only decreased by 7.4%, 8.6%, and 3.0%, respectively, after 1,000 bending cycles at a speed of 500 mm min^−1^ and a bending radius of approximately 2.5 mm, indicating the good mechanical flexibility and electrical stabilities of the TaS_2_ freestanding and composite films.

The XRD patterns further show that the TaS_2_ freestanding film exhibits a well-ordered lamellar structure (Figs. 5e and S19), and the corresponding interlayer distance (*d*) values listed in Table S5. The TaS_2_ freestanding film shows a strong XRD 2θ peak at 9.47° and a weak peak at 18.77°, corresponding to *d* of 0.933 and 0.472 nm, respectively. The *d* of the restacked TaS_2_ freestanding film increased compared with that in the TaS_2_ crystal, resulting in a significant reduction in the interlayer coupling. The fracture morphology of the TaS_2_ freestanding film shows a smooth curvature of 2H-TaS_2_ nanosheets (Fig. 5f), verifying high flexibility in the TaS_2_ freestanding films. Additionally, the bond-free vdW interfaces with large lateral dimensions allow adjacent TaS_2_ nanosheets to slide or rotate against each other to accommodate local structural perturbations (tension or compression) and reduce the strain-induced cracks and fractures without breaking the broad-area vdW interfaces and conduction pathways. (Fig. 5g) Therefore, the TaS_2_ freestanding film shows high flexibility as well as mechanical and electrical stability even under large deformations.

Compared with the TaS_2_ freestanding film, the introduction of protons during the preparation of the TaS_2_ composite films can significantly strengthen the interlayer interactions and induce the densification of the composite films. Thus, the TaS_2_/BC (10:5) and TaS_2_/ANFs (10:5) composite films show strong XRD peaks at 2θ of 14.78° and 14.66°, which correspond to *d* of 0.599 and 0.604 nm, respectively. These* d* values are smaller than that of the pure TaS_2_ freestanding film. The introduction of BC and ANFs nanofibers act as stiffeners because they are tightly embedded between the TaS_2_ nanosheets interlayer (Figs. 5f and S20), which effectively improves the flexibility, tensile strength, and toughness of the composite films. Figure 5h reveals the synergetic toughening mechanism of TaS_2_ composite films, which are attributed to the interfacial interaction (hydrogen bonding) between the fibers, van der Waals interaction between the TaS_2_ nanosheets, electrostatic interaction between fibers and bond-free TaS_2_ nanosheets, and mechanical entanglement. When the stretching procedure starts, the TaS_2_ nanosheets first slide past each other because of the weak vdW interactions. Meanwhile, the nanofibers crosslinking with TaS_2_ nanosheets via electrostatic interactions are stretched and further arrest crack propagation for accommodating large deformation before complete fracture of the sheets.

### Electrical Conductivity and EMI Shielding Performances of TaS_2_ Films

**Fig. 6:**
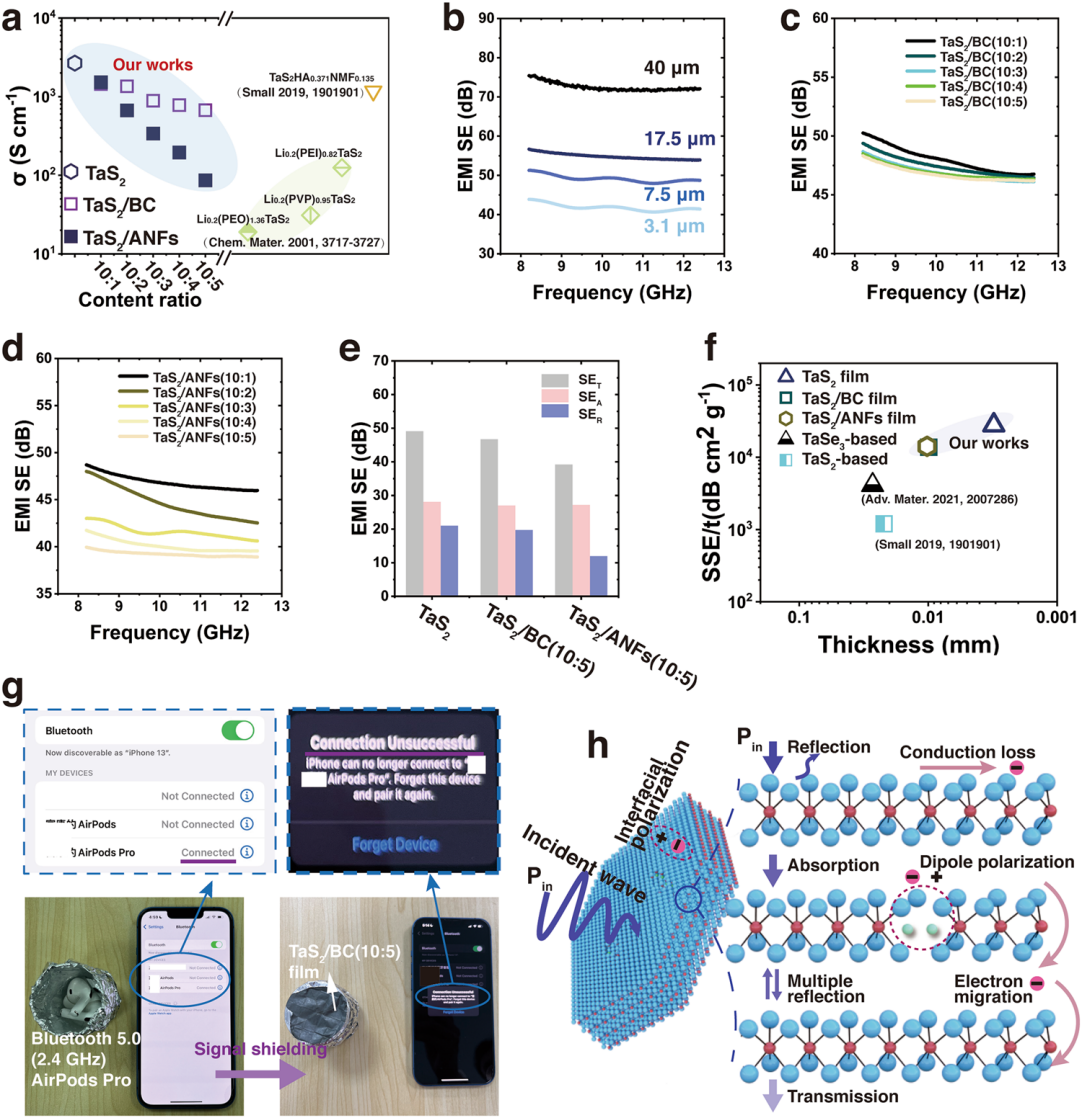
Electrical conductivity and EMI SE of TaS_2_ films. **a** Electrical conductivity of TaS_2_ freestanding film, TaS_2_/BC, and TaS_2_/ANFs composite films. **b** EMI SE of TaS_2_ freestanding films at different thicknesses. EMI SE of **c** TaS_2_/BC and **d** TaS_2_/ANFs composite films. **e** Average EMI SE_T_, SE_A_, and SE_R_ in 7.5-μm-thick TaS_2_ freestanding film, TaS_2_/BC (10:5), and TaS_2_/ANFs (10:5) composite films. (Note: TaS_2_ content is the same for each tested sample). **f** Comparison of EMI SSE/t of TaS_2_ films with the reported TMDs-based EMI shielding materials. **g** Demonstration of EMI shielding performance of TaS_2_/BC (10:5) composite film. **h** Schematic illustration of the proposed EMI shielding mechanism of the TaS_2_ films for ultra-high EMI SE

The electrical conductivity of the 3.1-μm-thick TaS_2_ freestanding film is 2666 S cm^−1^ (Fig. 6a and Table S6), which is significantly higher than that of the TaS_2_ powder (Fig. S21) and the reported TaS_2_ based films (Table S7). This result indicates that the broad-area dangling-bond-free plane-to-plane contacts in the TaS_2_ nanosheets along with their minimum interfacial trapping states and low voids can facilitate the in-plane and intersheet electron transport properties of the thin film [43, 63–65]. In addition, the presence of surface defects in the TaS_2_ nanosheets provides vertical paths for electron transmission and a passage for the adsorbed Li-ions, resulting in a high conductivity of the film [66–68]. The conductivity of the TaS_2_ composite films (Fig. 6a and Table S6) decreases with the increase in the BC or ANFs content due to the insertion of insulating fibers into the TaS_2_ nanosheet interlayer. The effect of ANFs on the conductivity is more prominent than that of BC because ANFs have smaller diameter (Fig. S9) and larger volume distribution (Fig. S6). However, the conductivity of the TaS_2_ composite films are superior to those of most reported TaS_2_-based films (Table S7). The alternating (between TaS_2_ and the fibers) multilayer stack structures of the TaS_2_ composite films ensured their optimal conductivity and mechanical properties.

In Fig. 6b, the ultra-high electrical conductivity of the 3.1-μm-thick TaS_2_ freestanding film resulted in an excellent EMI SE of 41.8 dB at the X-band (8.2–12.4 GHz), which is much higher than the commercialization benchmark (20 dB) in electronic equipment of civil telecom. Moreover, the EMI SE of the individual TaS_2_ films with a thickness of 7.5, 17.5, and 40 μm is 49.1, 54.8, and 72.5 dB, respectively, and hence, EMI SE increases with the increase in the thickness. In addition, the multi-level superimposed TaS_2_ films show superior EMI SE (Fig. S22). Thus, EMI SE increases with the increase in the number of layers and the film thickness. The multi-level superimposed TaS_2_ films (5P, ~ 52 μm) provides a superior EMI SE of 105.2 dB. Clearly, the superimposed film has better EMI SE performance than the independent film, which may be due to the synergistic effect of multiple internal reflections between the adjacent TaS_2_ films and multiple-wave interference between the TaS_2_ nanosheets [69–72]. The EMI SE of the TaS_2_/BC and TaS_2_/ANFs composite films were measured at different component ratios at the X-band. With the increase in the BC content, the EMI SE does not significantly decrease (Fig. 6c and Table S6). At a high BC content in the TaS_2_/BC (10:5) composite film, the average EMI SE still reaches 46.8 dB, which can effectively shield against a 2.4 GHz Bluetooth signal (Fig. 6g). This is highly satisfactory EMI SE for certain industrial applications. Among the TaS_2_/ANFs composite films (Fig. 6d and Table S6), TaS_2_/ANFs (10:1) achieved an excellent EMI SE of 46.8 dB. With the increase in the ANFs content, the EMI SE of the TaS_2_/ANFs (10:5) decreases to 39.2 dB, which can be mainly attributed to the significant decrease in its conductivity due to the ANFs. The main electromagnetic shielding mechanism of these films is reflection (Figs. 6e and S23), which is due to the high electrical conductivity of the lamellar restacked structure of TaS_2_ and the multilayer stack structures of the TaS_2_ composite films [7, 8, 58, 70]. Moreover, the absolute effectiveness (SSE/t) was used to evaluate the shielding performance of the TaS_2_ films considering the effects of density and thickness [73]. The SSE/t of the 3.1-μm-thick TaS_2_ freestanding film is 27,859 dB cm^2^ g^−1^ (Fig. 6f and Table S6), which is the highest value for TMD-based materials. The developed TaS_2_ freestanding film is also comparative to other materials such as graphene and MXene materials (Table S8). The excellent EMI SE performance of the TaS_2_ freestanding and composite films are ascribed to the stacked structure and defects of the TaS_2_ nanosheets and the multi-interfaces created by BC or ANFs, which synergistically contributed to a strong interfacial polarization as well as multiple reflections and increased the dielectric loss of the incident electromagnetic waves (Fig. 6h).

## Conclusions

2H-TaS_2_ nanosheets were successfully batch-produced using an environmentally friendly Li-ion solution-intercalated strategy, leading to a fast electron transmission and excellent mechanical properties of the restacked TaS_2_ films. The 3.1 μm-thick TaS_2_ freestanding film exhibits an ultra-high electrical conductivity of 2666 S cm^−1^, an excellent EMI SE of 41.8 dB, a recorded SSE/t of 27,859 dB cm^2^ g^−1^, and a high tensile strength of 23.3 ± 4.8 MPa. This combination of electrical and mechanical properties originates from the vdW interactions among the staggered 2H-TaS_2_ nanosheets, allowing natural interfacial strain relaxation and accommodating local structural perturbation in the freestanding film. Furthermore, the TaS_2_ composite films exhibit excellent EMI shielding properties and higher tensile strength with better mechanical flexibility. This study can be used as a basis for similar research on the large family of TMDs with widely tunable electrical and mechanical properties, which is promising for applications in the fields of EMI shielding and nanodevices that mainly rely on 2D materials.

### Supplementary Information

Below is the link to the electronic supplementary material.

Supplementary file1 (PDF 2865 KB)

Supplementary file2 (MP4 16198 KB)

Supplementary file3 (MP4 2462 KB)

Supplementary file4 (MP4 15097 KB)

Supplementary file5 (MP4 14082 KB)
