# Supplementary material for: Regulating the Electrical and Mechanical Properties of TaS2 Films via van der Waals and Electrostatic Interaction for High Performance Electromagnetic Interference Shielding
Source: Nanomicro Lett. 2023 Apr 18;15:106. doi: 10.1007/s40820-023-01061-1 (PMC10113419; doi:10.1007/s40820-023-01061-1)
Supplement: Supplementary file 1 — Supplementary file1 (PDF 2865 KB) [file 40820_2023_1061_MOESM1_ESM.pdf]

Supporting Information for

Regulating the Electrical and Mechanical Properties of TaS_2 Films via van der Waals and Electrostatic Interaction for High Performance Electromagnetic Interference Shielding

Fukang Deng[1], Jianhong Wei[1, 2], Yadong Xu[1], Zhiqiang Lin[1], Xi Lu[1], Yan-Jun Wan[1], Rong Sun[1, *], Ching-Ping Wong[3], and Yougen Hu[1, *]

[1] Shenzhen Institute of Advanced Electronic Materials, Shenzhen Institute of Advanced Technology, Chinese Academy of Sciences, Shenzhen 518055, P. R. China

[2] Shenzhen Geim Graphene Center, Institute of Materials Research, Shenzhen International Graduate School, Tsinghua University, Shenzhen 518055, P. R. China

[3] School of Materials Science and Engineering, Georgia Institute of Technology, Atlanta, GA 30332, USA

*Corresponding authors. E-mail: yg.hu@siat.ac.cn (Yougen Hu); rong.sun@siat.ac.cn (Rong Sun)

Supplementary Figures and Tables

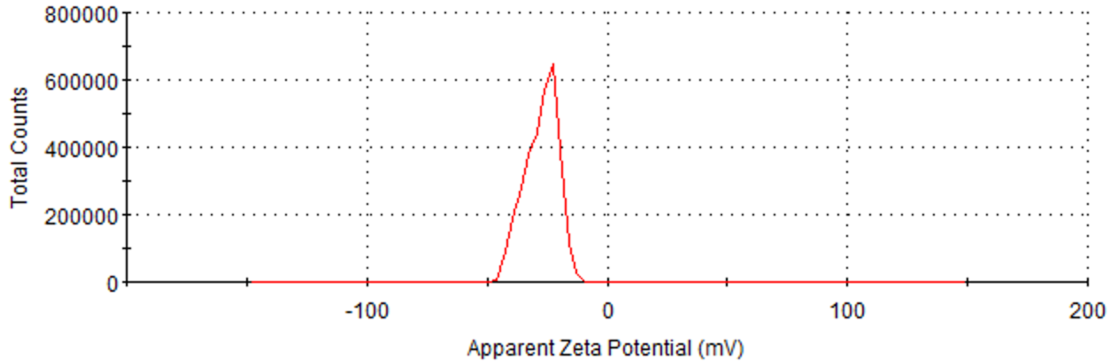

Fig. S1 The Zeta potential of TaS_2 nanosheets aqueous dispersions (~0.1 mg mL^{-1}), showed a value of -27.4 mV

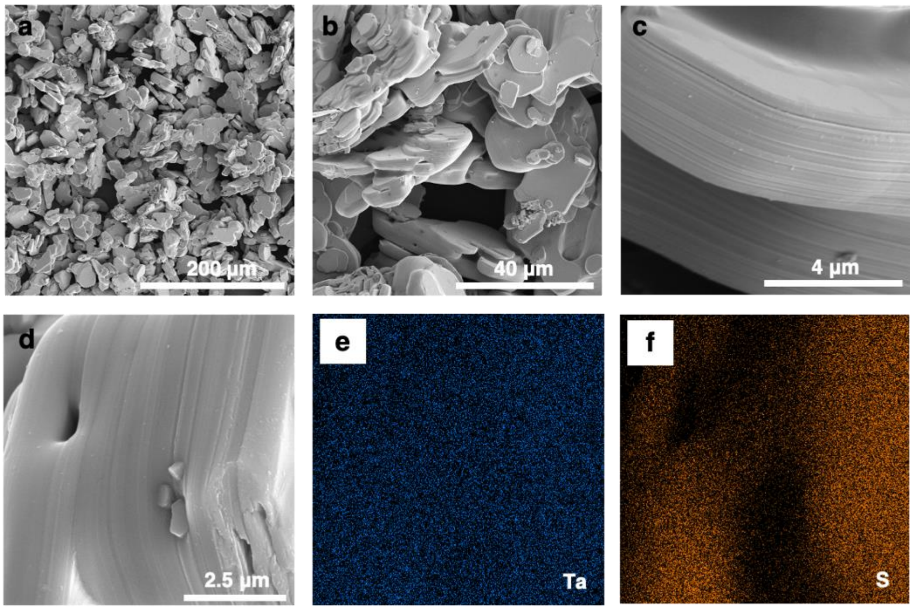

Fig. S2 a-d SEM images of bulk 2H-TaS_2. **e**, **f** EDS elemental mapping of bulk 2H-TaS_2.

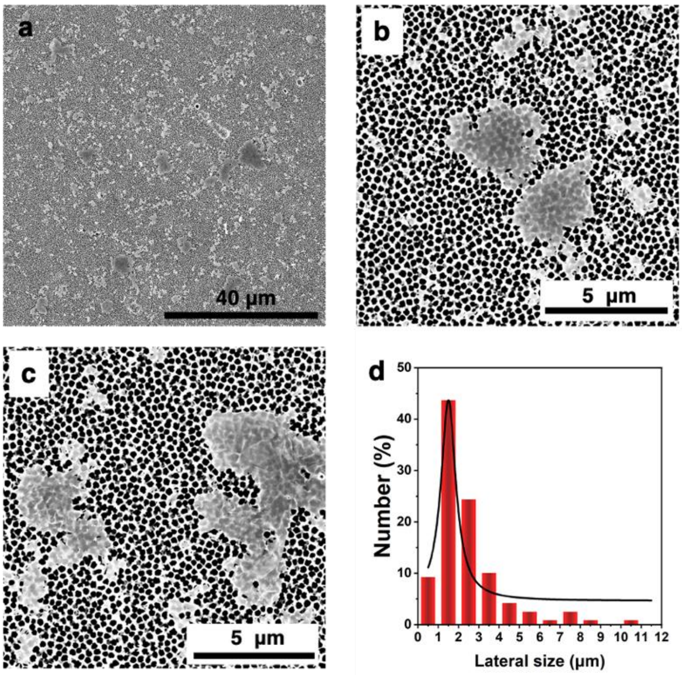

Fig. S3 Calculation of lateral size of 2H-TaS_2 nanosheets. **a-c** SEM images of 2H-TaS_2 nanosheets. **d** Lateral size distribution of 2H-TaS_2 nanosheets indicates that the average lateral size of 2H-TaS_2 nanosheets is roughly 1.5 µm

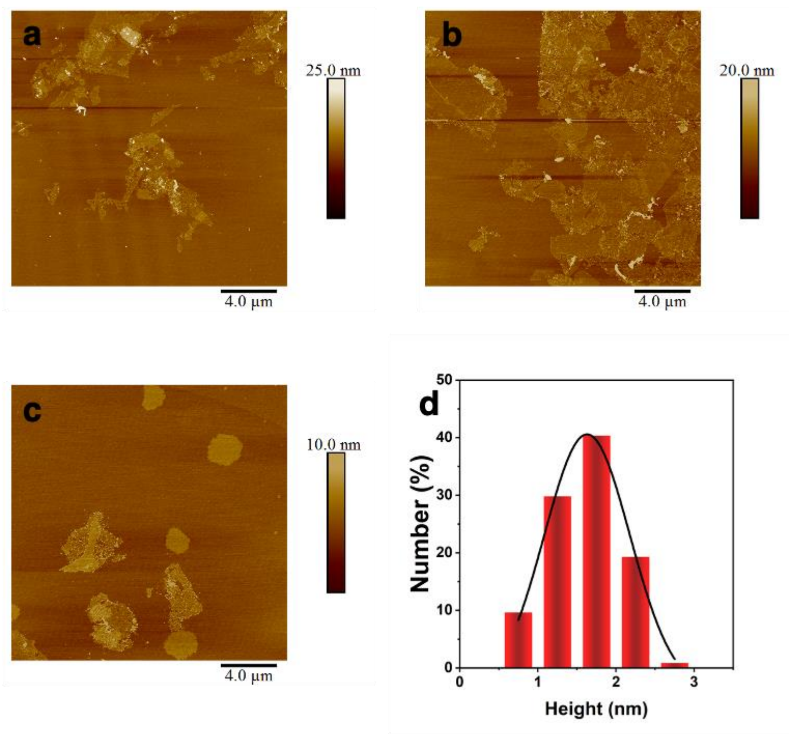

Fig. S4 Thickness of 2H-TaS_2 nanosheets. **a**-**c** AFM images of 2H-TaS_2 nanosheets. **d** Height distribution of 2H-TaS_2 nanosheets, indicating that the average thickness of 2H-TaS_2 nanosheets is roughly 1.74 nm

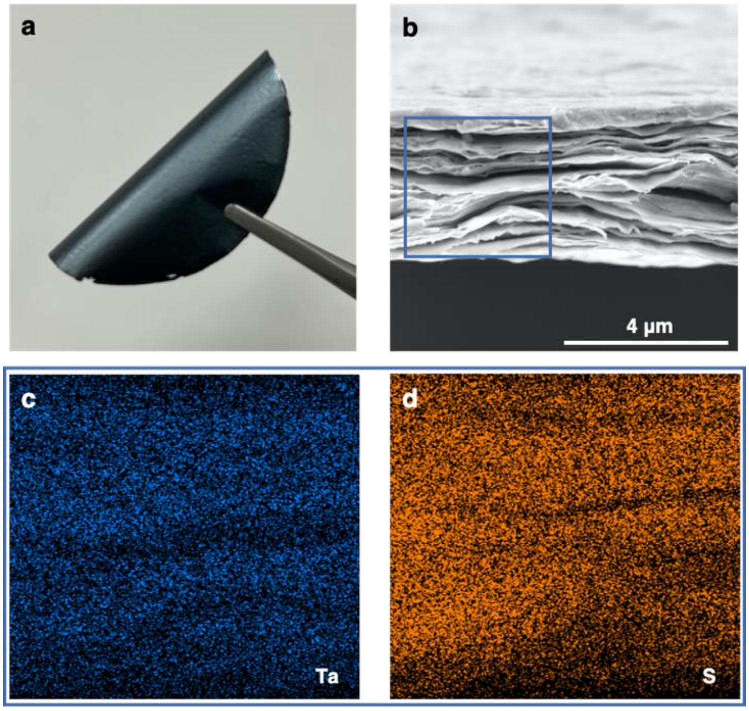

Fig. S5 Morphology of TaS_2 freestanding film. **a** Photograph of TaS_2 freestanding film exhibiting its flexibility. **b** SEM image of the fracture surface of the TaS_2 freestanding film. **c**, **d** EDS mapping of elements Ta and S for the area outlined in figure S5b

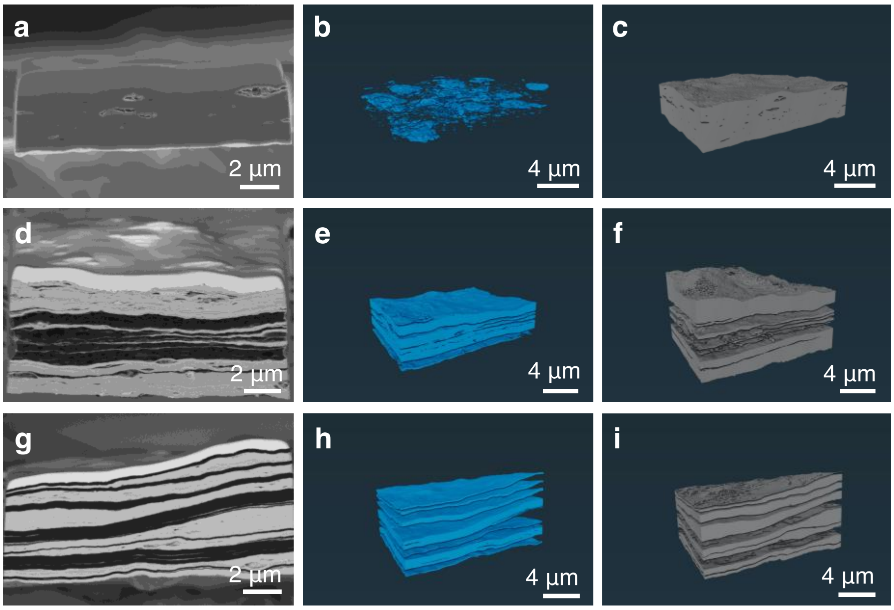

Fig. S6 FIB/SEMT cross-sectional of TaS_2, TaS_2 /BC (10:1), and TaS_2/ANFs (10:1) films. A cross-sectional derived from FIB/SEMT for **a** TaS_2 freestanding film, **d** TaS_2/BC (10:1) composite film, and **g** TaS_2/ANFs (10:1) composite film. The volumes of **b** voids and **c** TaS_2 derived from FIB/SEMT for TaS_2 freestanding film are 33.8162 μm^3 and 563.087 μm^3 respectively, with a porosity of 6.01%. The volumes of **e** BC and **f** TaS_2 derived from FIB/SEMT for TaS_2/BC (10:1) composite film are 410.809 μm^3 and 1334.81 μm^3 respectively, with a volume rate of 30.78%. The volumes of **h** ANFs and **i** TaS_2 derived from FIB/SEMT for TaS_2 /ANFs (10:1) composite film are 640.916 μm^3 and 1609.96 μm^3 respectively, with a volume rate of 39.81%

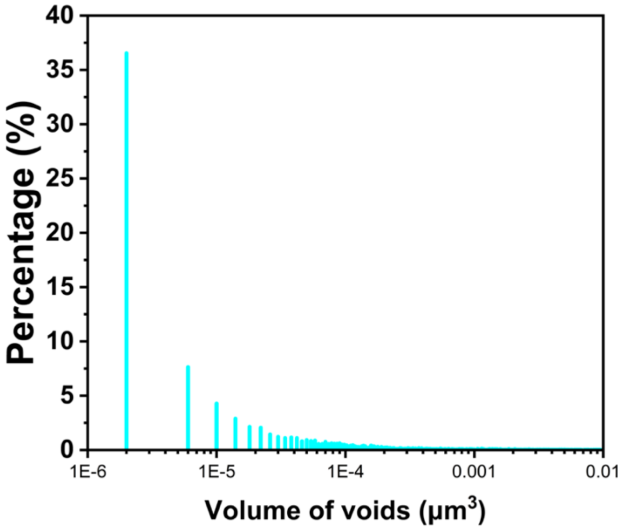

Fig. S7 Volume distribution of 3D reconstructed voids derived from FIB/SEMT for TaS_2 freestanding film

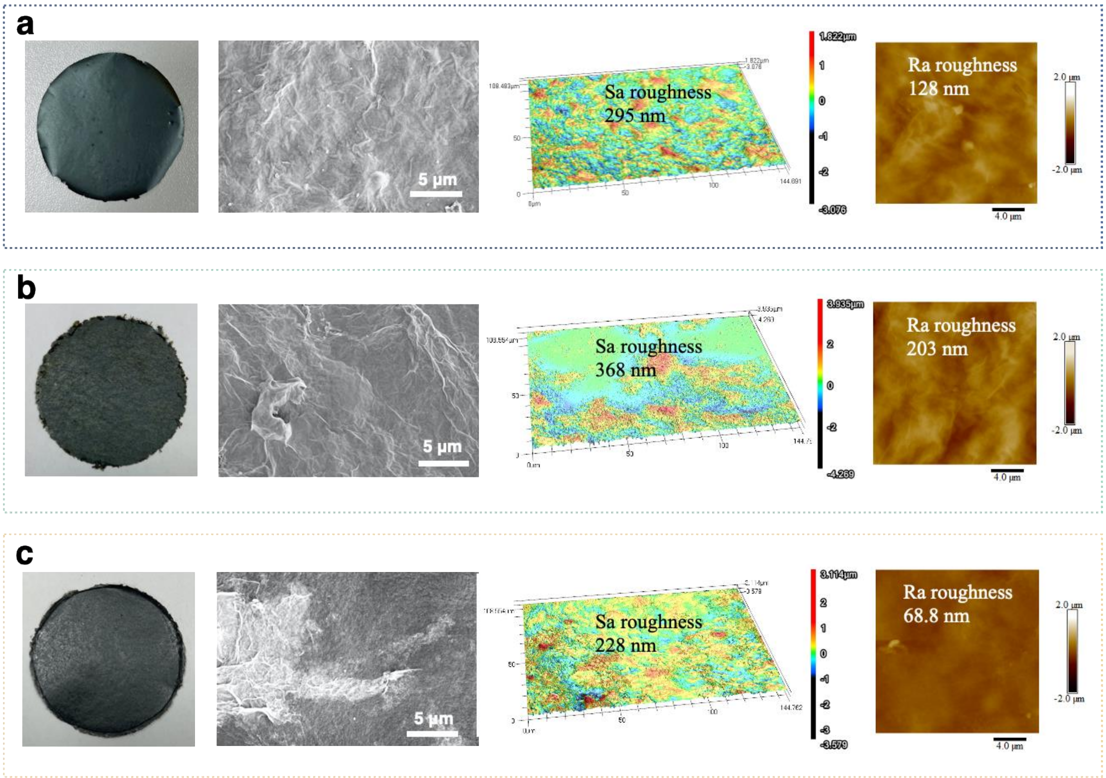

Fig. S8 Digital photographs and SEM, 3D, and AFM images for the top surface for **a** TaS_2 freestanding film, **b** TaS_2/BC (10:5) composite film, **c** TaS_2/ANFs (10:5) composite film

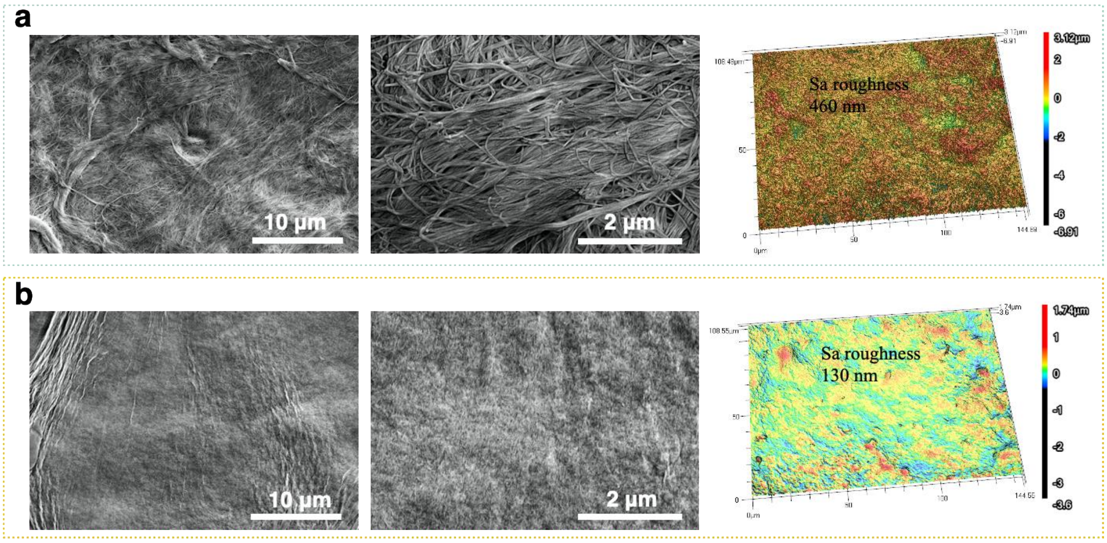

Fig. S9 SEM and 3D images for the top surface for **a** the pure BC and **b** ANFs films

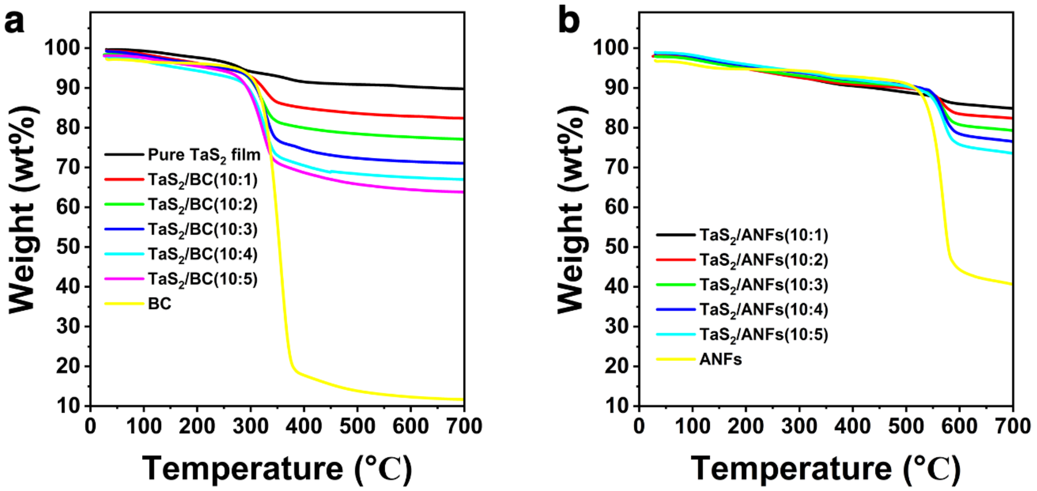

Fig. S10 TGA curves. **a** TaS_2 freestanding film and TaS_2/BC composite films **b** TaS_2/ANFs composite films. The TGA characterizations were performed from 30 to 700 ℃ at a temperature rising rate of 10 ℃/min under a nitrogen atmosphere

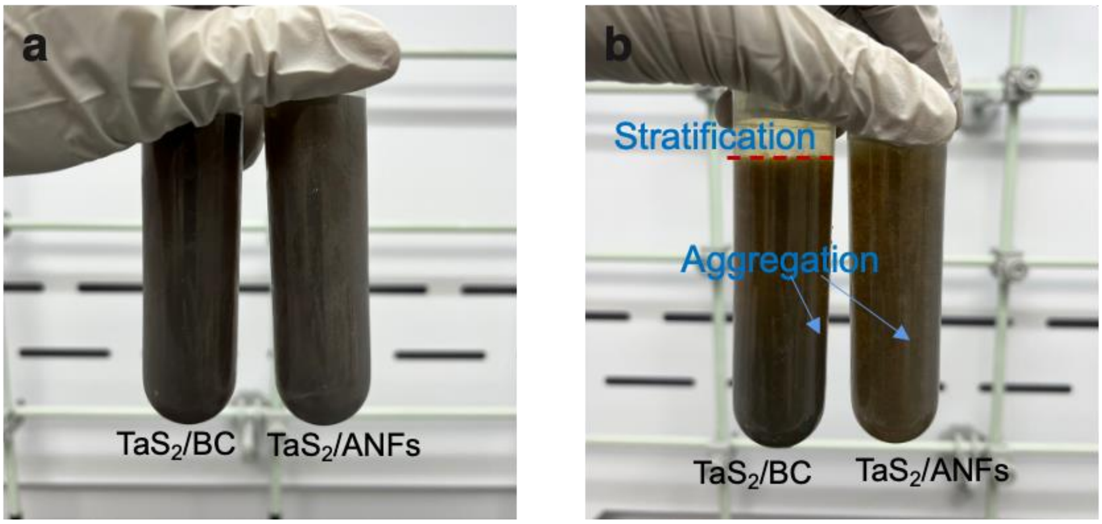

Fig. S11 The images of TaS_2/BC and TaS_2/ANFs dispersion **a** before and **b** after acid treatment

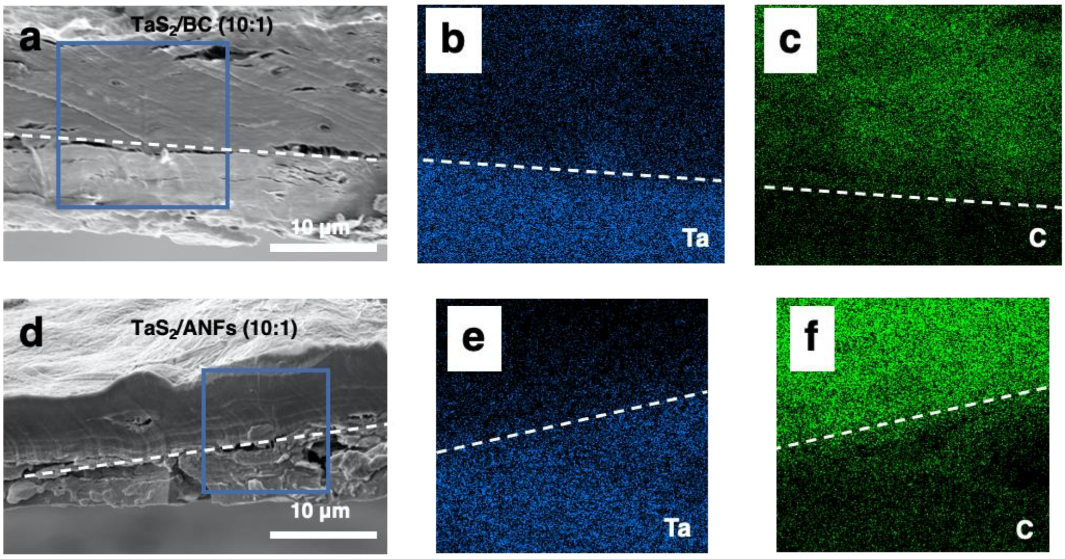

Fig. S12 Morphology of TaS_2/BC and TaS_2/ANFs films without acid treatment

SEM image of the section of **a** the TaS_2/BC and **d** TaS_2/ANFs films. Corresponding EDS mapping of elements Ta and C for the area outlined in **a**, **d** for **b**, **c** TaS_2/BC and **e**, **f** TaS_2/ANFs composite films.

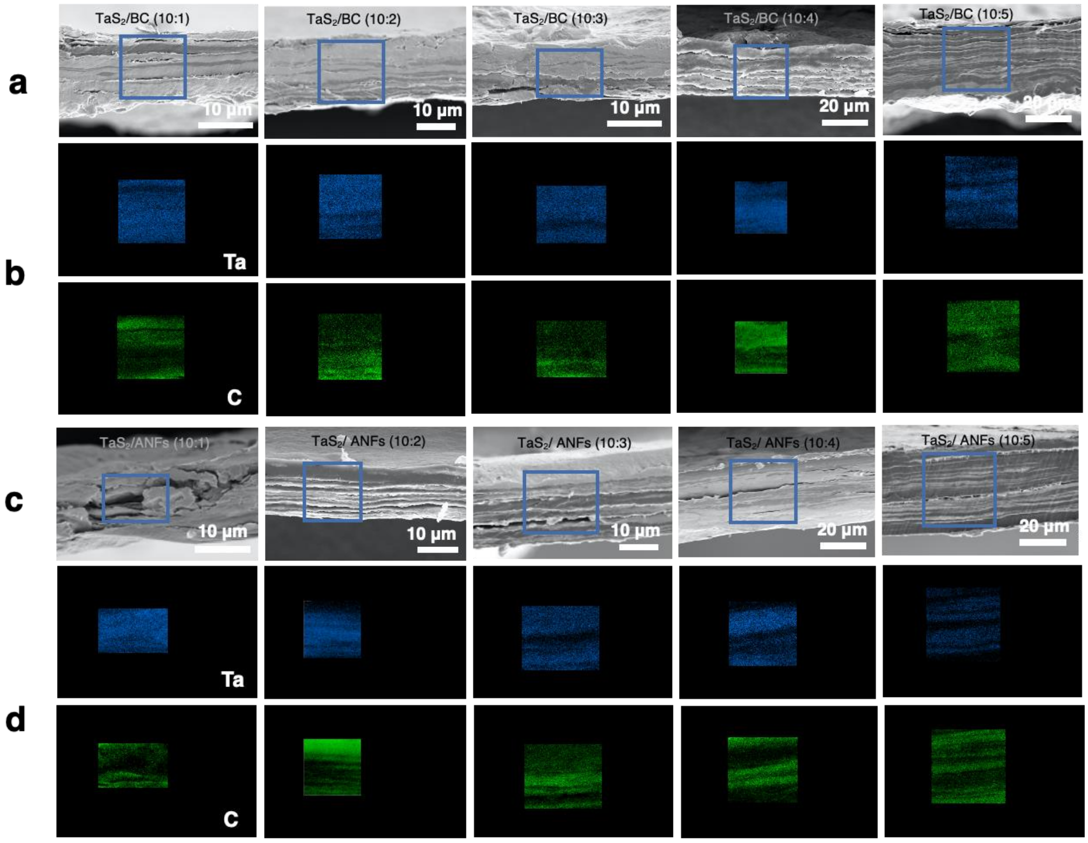

Fig. S13 Morphology of TaS_2/BC and TaS_2/ANFs composite films with HCl treatment. SEM images of the section of the **a** TaS_2/BC and **c** TaS_2/ANFs films. Corresponding EDS mapping of elements Ta and C for the area outlined in **a**, **c** for **b** TaS_2/BC and **d** TaS_2/ANFs films

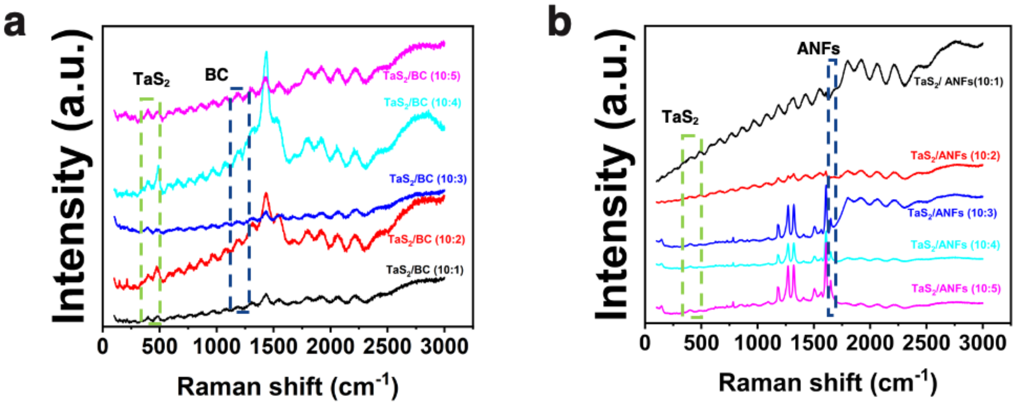

Fig. S14 Raman spectra of **a** TaS_2/BC and **b** TaS_2/ANFs composite films

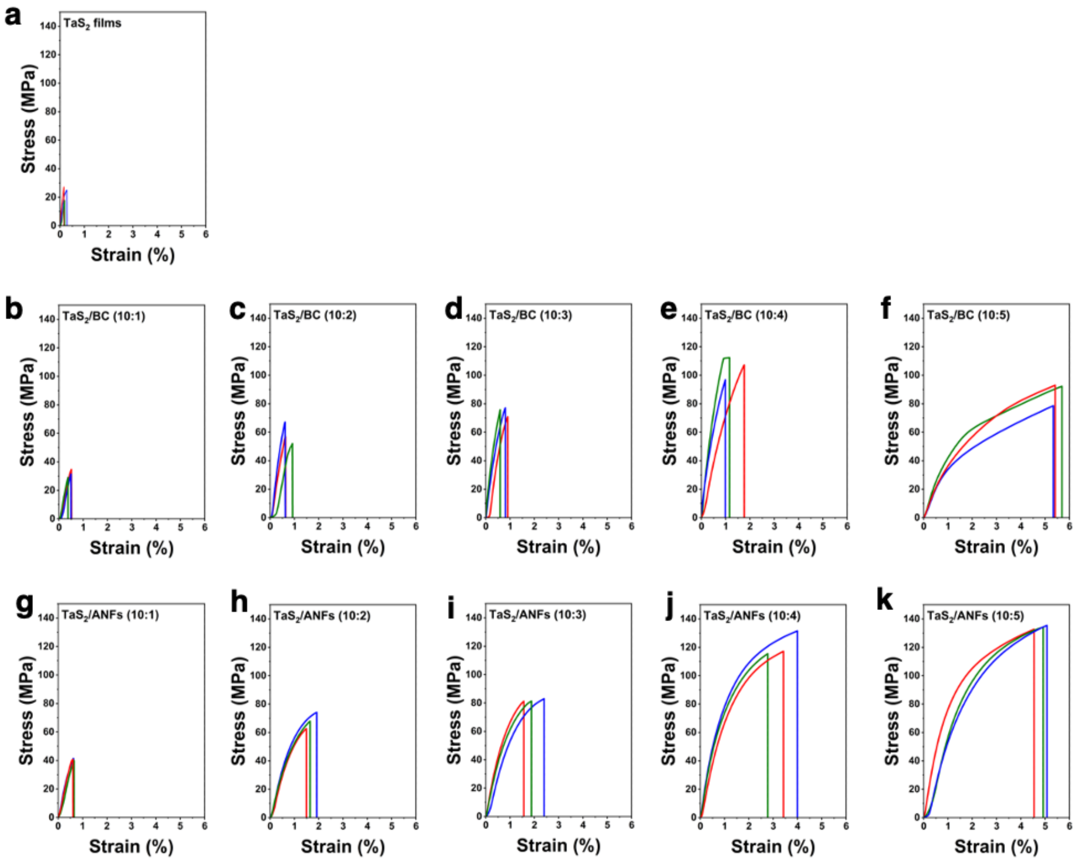

Fig. S15 Tensile stress–strain curves of the films. **a** TaS_2 freestanding film, **b-f** TaS_2/BC (10:1) to TaS_2/BC (10:5) composite films, **g-h** TaS_2/ANFs (10:1) to TaS_2/ ANFs (10:5) composite films

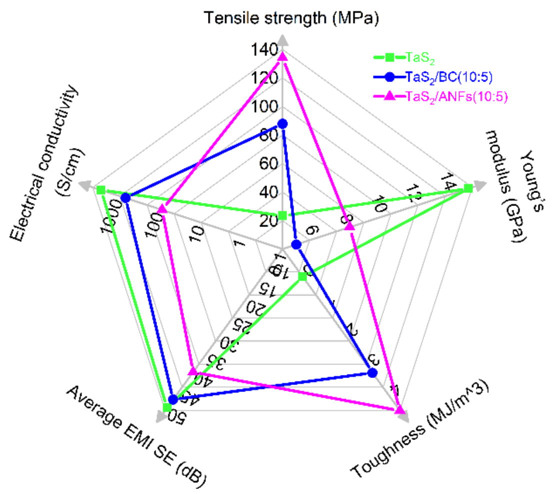

Fig. S16 A radial plot comparing the tensile strength, Young's modulus, toughness, electrical conductivity, and EMI SE of TaS_2 freestanding film, TaS_2/BC (10:5) composite film, and TaS_2/ANFs (10:5) composite film

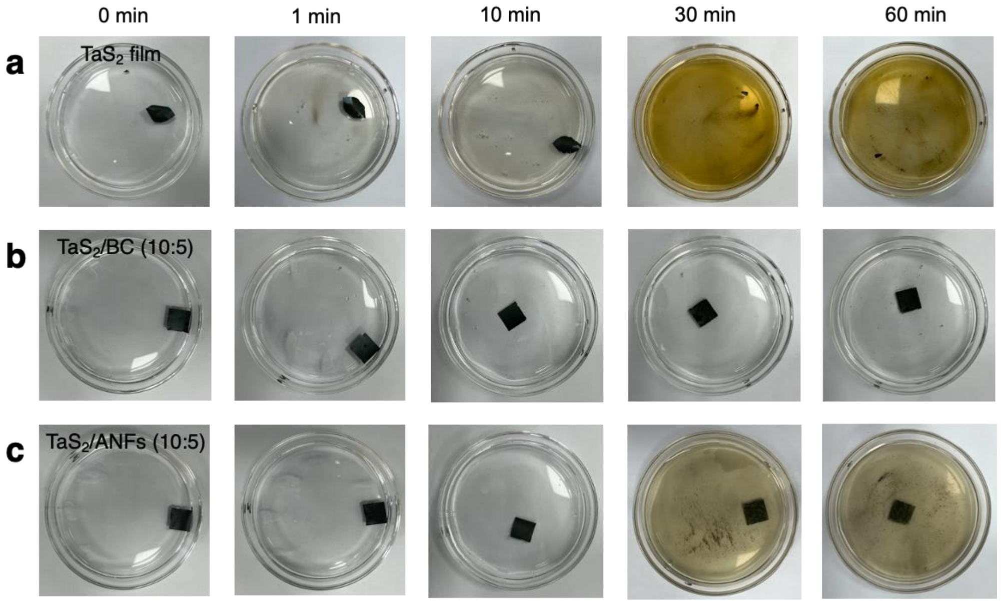

Fig. S17 Photographs of the films during ultrasonication (using a 500 W, 40 kHz sonicator) in water. **a** TaS_2 freestanding film, **b** TaS_2/BC (10:5) composite film, **c** TaS_2/ANFs (10:5) composite film. The TaS_2 freestanding film, TaS_2/BC (10:5) composite film, and TaS_2/ANFs (10:5) composite film begin to disintegrate after ultrasonication for 1, 30, and 30 min, respectively

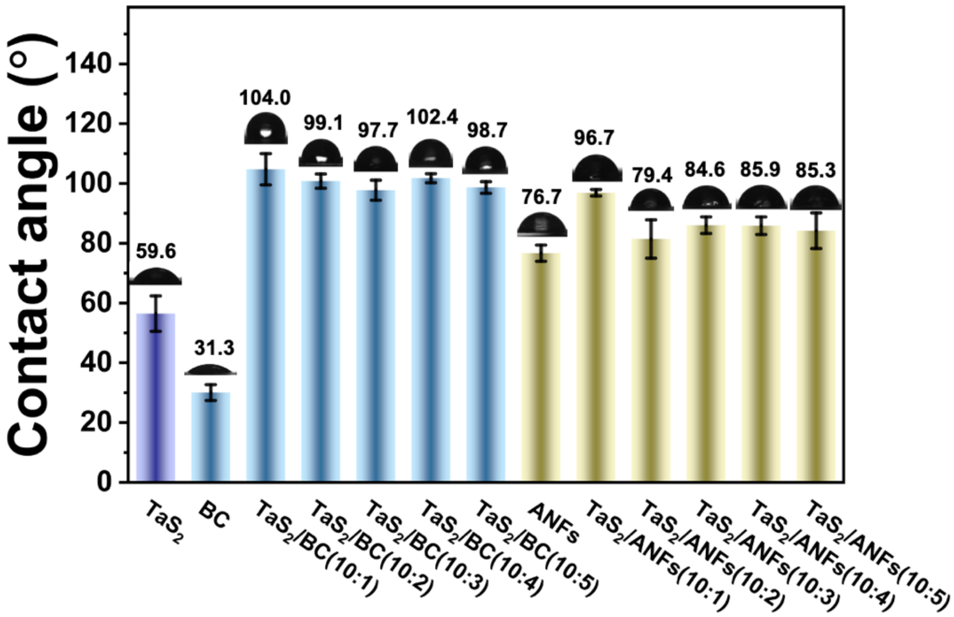

Fig. S18 Wettability of TaS_2 freestanding film, TaS_2/BC composite films, and TaS_2/ANFs composite film toward water. The possible reason for the increase of the contact angle of the composite films is the change of the films surface structure

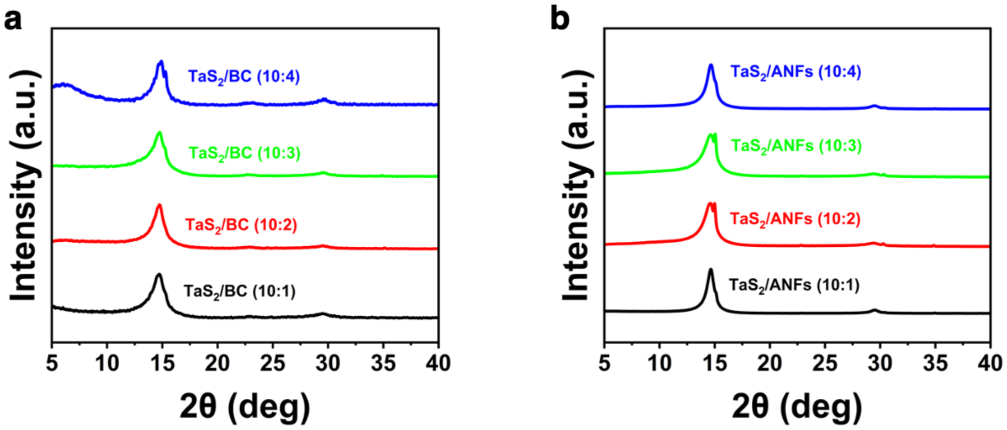

Fig. S19 XRD curves using Cu-Kα radiation. Normalized XRD patterns of **a** TaS_2/BC composite films and **b** TaS_2/ANFs composite films, Zoom-in of the XRD patterns in the range of 5-40 deg

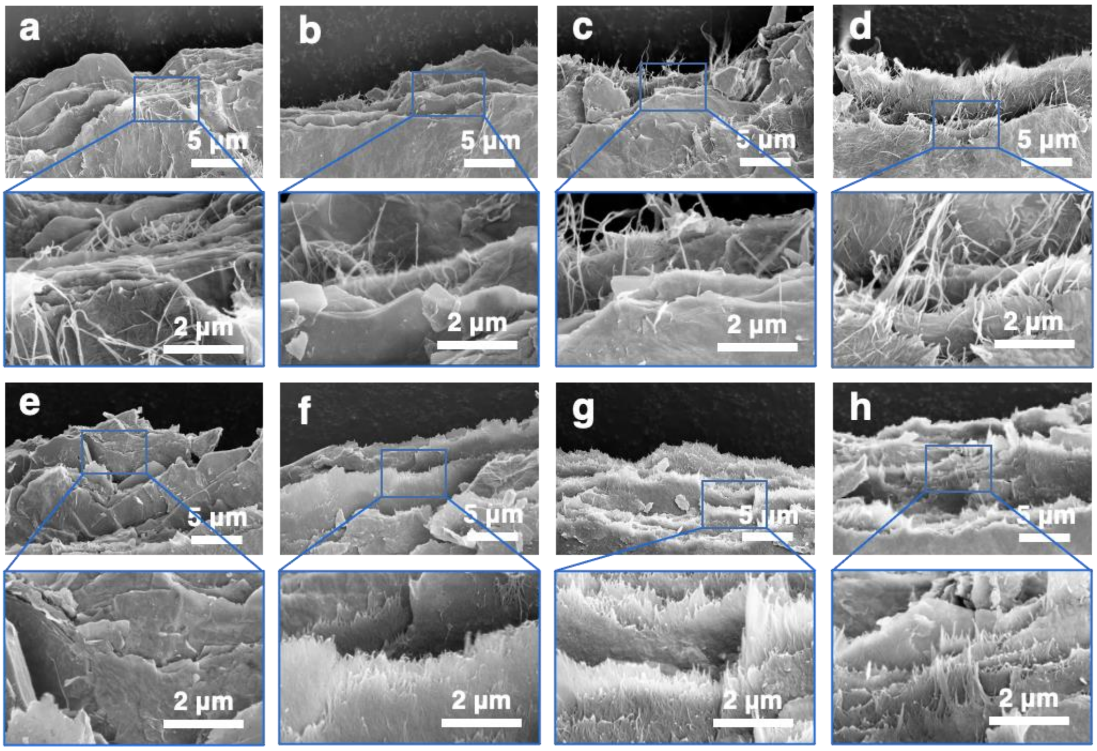

Fig. S20 Inclined-view SEM images of the fracture surface of the composite films. **a-d** TaS/BC (10:1) to (10:4), and **e-h** TaS_2/ANFs (10:1) to (10:4). The TaS_2/BC and TaS_2/ANFs films have fiber distribution at the fracture edge

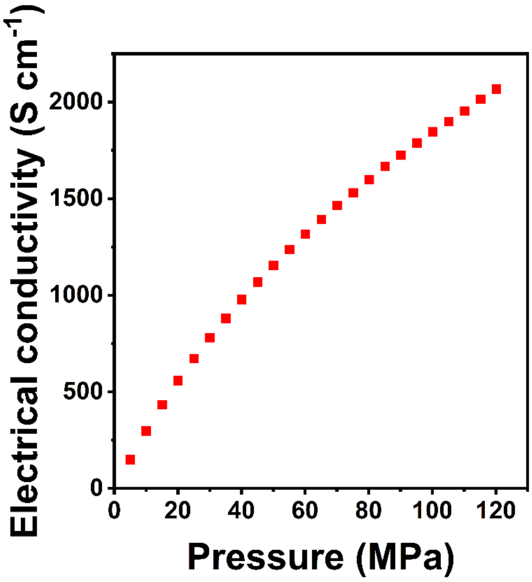

Fig. S21 The electrical conductivity of 2H-TaS_2 powder corresponding to the pressure. The electrical conductivity of 2H-TaS_2 powder increases with the increase of pressure and reaches 2,067 S cm^{-1} at a high pressure of 120 MPa

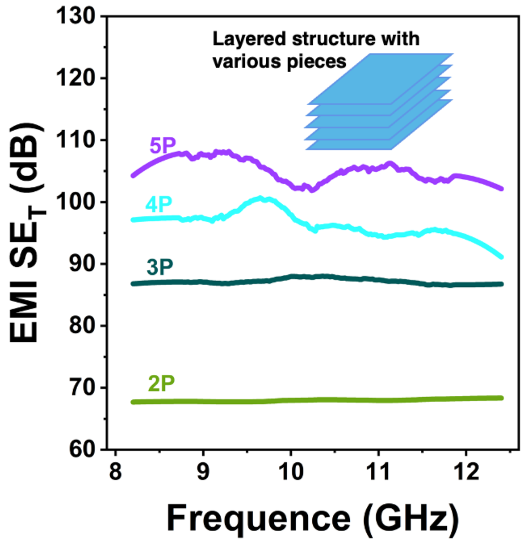

Fig. S22 EMI SE of the multi-level superimposed of TaS_2 films. 2P (~16 μm) indicated the superposition of two 8-μm-thick TaS_2 films; 3P (~36 μm) indicated the superposition of 10-μm-thick and two 13-μm-thick TaS_2 films; 4P (~42 μm) indicated the superposition of two 8-μm-thick and two 13-μm-thick TaS_2 films; 5P (~52 μm) indicated the superposition of two 8-μm-thick and 10-μm-thick and two 13-μm-thick TaS_2 films. The average EMI SE of TaS_2 film thickness of 2P, 3P, 4P, and 5P are 68.0 dB, 87.2 dB, 96.4 dB, and 105.2 dB respectively

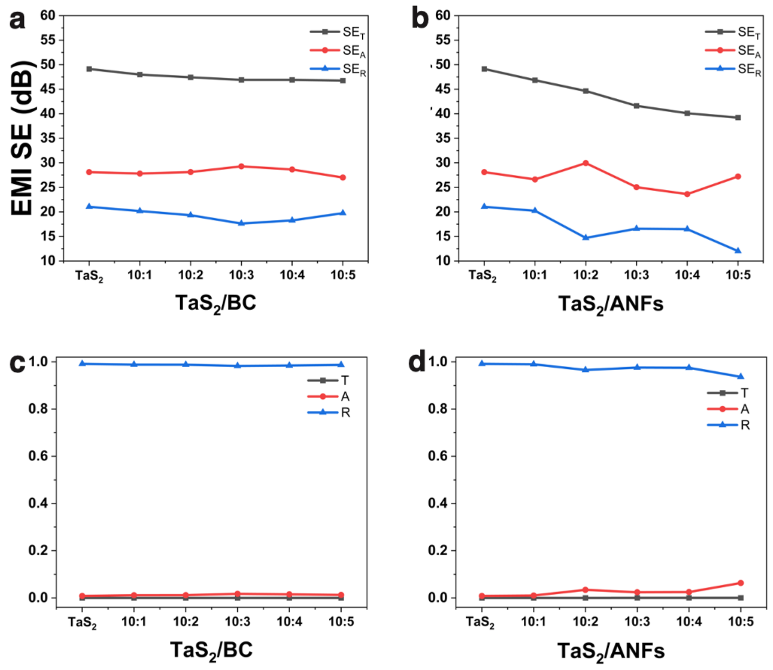

Fig. S23 a, **b** EMI SE_T, SE_A, and SE_R of 7.5-μm-thick TaS_2 film, TaS_2/BC composite film, and TaS_2/ANFs composite film. **c**, **d** The transmission (T), absorption (A), and reflection (R) coefficient of 7.5-μm-thick TaS_2 film, TaS_2 /BC composite film, and TaS_2 /ANFs composite film. (Note: TaS_2 content is the same)

Table S1 Chemical compositions of the $Li_xTa_yS_z$ nanosheets

	x	y	z	Chemical formula
$Li_xTa_yS_z$	0.051599	0.287759	0.592451	$Li_{0.18}TaS_2$

Table S2 BC and ANFs content in TaS_2/BC and TaS_2/ANFs composite films, determined by TGA measurements

Sample	BC content (wt%)	ANFs content (wt%)
TaS_2/BC (10:1)	9.44	-
TaS_2/BC (10:2)	16.21	-
TaS_2/BC (10:3)	23.95	-
TaS_2/BC (10:4)	29.15	-
TaS_2/BC (10:5)	33.22	-
TaS_2/ANFs (10:1)	-	5.27
TaS_2/ANFs (10:2)	-	15.06
TaS_2/ANFs (10:3)	-	21.27
TaS_2/ANFs (10:4)	-	26.91
TaS_2/ANFs (10:5)	-	33.01

Table S3 Thinkness and mechanical properities of the TaS_2 freestanding, TaS_2/BC and TaS_2/ANFs films

Sample	Thickness (μm)	Tensile strength (MPa)	Strain to failure (%)	Young's modulus (GPa)	Toughness (MJ m^{-3})
TaS_2 film	3.1	23.3±4.8	0.21±0.07	14.9±6.2	0.033±0.018
TaS_2/BC (10:1)	9.9	31.7±2.8	0.45±0.08	8.7±0.9	0.066±0.017
TaS_2/BC (10:2)	10.2	58.7±7.7	0.72±0.17	12.9±3.8	0.19±0.02
TaS_2/BC (10:3)	15.6	74.5±3.3	0.76±0.16	15.5±2.4	0.31±0.05
TaS_2/BC (10:4)	17.8	105.5±7.9	1.29±0.41	14.0±4.8	0.83±0.25
TaS_2/BC (10:5)	24.18	87.9±8.1	5.48±0.19	4.9±0.6	3.25±0.45
TaS_2/ANFs (10:1)	10.0	40.7±0.8	0.63±0.02	8.09±0.31	0.134±0.009
TaS_2/ANFs (10:2)	13.3	68.2±5.8	1.68±0.22	7.11±0.43	0.72±0.19
TaS_2/ANFs (10:3)	15.2	81.9±1.0	1.94±0.42	8.10±0.98	1.02±0.23
TaS_2/ANFs (10:4)	22.4	121.4±8.8	3.39±0.61	9.60±1.04	2.93±0.85
TaS_2/ANFs (10:5)	25.5	134.13±1.4	4.84±0.27	8.0±1.46	4.52±0.07

Table S4 The mechanical properities of the TaS_2-based films

**Sample**	**Tensile strength (MPa)**	**Strain to failure (%)**	**Young's modulus (MPa)**	**Toughness (MJ m^{-3})**	Refs.
$TaS_2HA_{0.371}NMF_{0.135}$	9.16	-	-	-	[S1]
PEO/TaS_2(0.5 wt%)	11.27±1.72	4.17±0.57	632.30±22.45	-	[S2]

Table S5 Interlayer diffraction spacing of TaS_2 freestanding film, TaS_2/BC and TaS_2/ANFs composite films

**Sample**	***d*** **(nm)**
TaS_2	0.933
TaS_2/BC (10:1)	0.599
TaS_2/BC (10:2)	0.600
TaS_2/BC (10:3)	0.600
TaS_2/BC (10:4)	0.600
TaS_2/BC (10:5)	0.599
TaS_2/ANFs (10:1)	0.604
TaS_2/ANFs (10:2)	0.604
TaS_2/ANFs (10:3)	0.605
TaS_2/ANFs (10:4)	0.604
TaS_2/ANFs (10:5)	0.604

Table S6 Thickness, density, sheet resistance, electrical conductivity, average EMI SE between 8.2 and 12.4 GHz, and absolute EMI SE (SSE/t) for the TaS_2 freestanding film, TaS_2/BC, and TaS_2/ANFs composite films

**Sample**	**Thickness (μm)**	**Density (g cm^{-3})**	**Sheet resistance (Ω sq^{-1})**	**Electrical conductivity (S cm^{-1})**	**Average EMI SE (dB)**	**SSE/t (dB cm^2 g^{-1})**
TaS_2	3.1	4.84	1.21	2666	41.8	27,859
TaS_2/BC (10:1)	9.9	3.52	0.704	1435	48.0	13,774
TaS_2/BC (10:2)	10.2	3.44	0.725	1352	47.4	13,509
TaS_2/BC (10:3)	15.6	2.41	0.724	886	46.9	12,527
TaS_2/BC (10:4)	17.8	2.58	0.723	777	46.9	10,213
TaS_2/BC (10:5)	24.18	2.56	0.617	670	46.8	7,560
TaS_2/ANFs (10:1)	10.0	3.30	0.659	1517	46.8	14,182

TaS_2/ANFs (10:2)	13.3	3.09	1.13	665	44.6	10,852
TaS_2/ANFs (10:3)	15.2	2.75	1.94	339	41.6	9,952
TaS_2/ANFs (10:4)	22.4	2.57	2.29	195	40.1	6,966
TaS_2/ANFs (10:5)	25.5	2.66	4.55	86	39.2	5,779

Table S7 Comparison on electrical conductivity of TaS_2 samples.

**Sample**	**Conductivity ($S\ m^{-1}$)**	**Refs.**
TaS_2	6.18×10^3	[S3]
$Pd_{0.1}TaS_2$	1.36×10^4	
single-crystal 2H-TaS_2	6.8×10^4	[S4]
2H-TaS_2 flakes	3.33×10^6 (in-plane)<br>12.5 (out-of-plane)	[S5]
$Li_{0.2}TaS_2$	$>10^5$	[S6]
$Li_{0.2}(PEO)_{1.36}TaS_2$	1,900	
$Li_{0.2}(PEO)_{1.68}TaS_2$	300	
$Li_{0.2}(PVP)_{0.95}TaS_2$	3,100	
$Li_{0.2}(PEI)_{0.82}TaS_2$	12,500	
$TaS_2HA_{0.371}NMF_{0.135}$	1.1738×10^5	[S1]
TaS_2 freestanding film	2.666×10^5	**This work**

Table S8 Thickness, EMI SE, and absolute EMI SE (SSE/t) of TaS_2-based films and other solid shielding films, including graphene, CNT, metal foils, MXene, TMTs, and TMDs

**Type**	**Materials**	**Thickness (mm)**	**EMI SE (dB)**	**SSE/t ($dB\ cm^2\ g^{-1}$)**	**Refs.**
Graphene-based	Graphene foam	0.027	68	14,309.8	[S7]
	Graphene film	0.0084	20	13,200	[S8]
	Graphene paper	0.05	62	18,235	[S9]
	Graphene membrane	1.5	38	70,371	[S10]
CNT-based	CNT	0.00185	51	199,100	[S11]
	CNT/Aramid Nanofiber	0.568	41.9	18,304.6	[S12]
	CNT/Graphene Edge hybrid foam	1.6	47	33,005.6	[S13]

	CNT sponge	1.8	54.8	30,444	[S14]
Mental-based	Al Foil	0.008	66	30,555	[S15]
	Cu Foil	0.001	70	7,812	
	AgNWs/EPM	1	111.5	13,433	[S16]
MXene-based	MXene film	0.011	68	25,863	[S15]
	MXene/SA	0.008	57	30,830	
	MXene	0.006	32	137,000	[S17]
	MXene	0.0034	60.9	60,309	[S18]
TMTs-based	SA/$TaSe_3$	0.027	21	4,115	[S19]
TMDs-based	TaS_2	0.022	31	~1,200	[S1]
	TaS_2 film	0.0031	41.8	27,859	**This work**

Supplementary References

[S1] P. A. Zong, D. Yoo, P. Zhang, Y. Wang, Y. Huang et al., Flexible foil of hybrid TaS_2 /organic superlattice: Fabrication and electrical properties. Small **16**(15), e1901901 (2020). https://doi.org/10.1002/smll.201901901

[S2] J. Shen, J. Wu, M. Wang, P. Dong, J. Xu et al., Surface tension components based selection of cosolvents for efficient liquid phase exfoliation of 2D materials. Small **12**(20), 2741-2749 (2016). https://doi.org/10.1002/smll.201503834

[S3] D. Wang, X. Wang, Y. Lu, C. S. Song, J. Pan et al., Atom-scale dispersed palladium in a conductive $Pd_{0.1}TaS_2$ lattice with a unique electronic structure for efficient hydrogen evolution. J. Mater. Chem. A **5**(43), 22618-22624 (2017). https://doi.org/10.1039/c7ta06447k

[S4] L. Li, X. Deng, Z. Wang, Y. Liu, M. Abeykoon et al., Superconducting order from disorder in 2H-$TaSe_{2-x}S_x$. npj Quantum Mater. **2**(1), (2017). https://doi.org/10.1038/s41535-017-0016-9

[S5] Y. Y. Liu, J. J. Wu, K. P. Hackenberg, J. Zhang, Y. M. Wang et al., Self-optimizing, highly surface-active layered metal dichalcogenide catalysts for hydrogen evolution. Nat. Energy **2**(9), 17127 (2017). https://doi.org/10.1038/nenergy.2017.127

[S6] L. Wang, M. G. Kanatzidis, Laminated TaS_2/polymer nanocomposites through encapsulative precipitation of exfoliated layers. Chem. Mater. **13**(10), 3717-3727 (2001). https://doi.org/10.1021/cm011025f

[S7] E. Zhou, J. Xi, Y. Liu, Z. Xu, Y. Guo et al., Large-area potassium-doped highly conductive graphene films for electromagnetic interference shielding. Nanoscale **9**(47), 18613-18618 (2017). https://doi.org/10.1039/c7nr07030f

[S8] B. Shen, W. T. Zhai, W. G. Zheng, Ultrathin flexible graphene film: An excellent thermal conducting material with efficient EMI shielding. Adv. Funct. Mater. **24**(28), 4542-4548 (2014). https://doi.org/10.1002/adfm.201400079

[S9] L. Zhang, N. T. Alvarez, M. X. Zhang, M. Haase, R. Malik et al., Preparation and characterization of graphene paper for electromagnetic interference shielding. Carbon **82**, 353-359 (2015). https://doi.org/10.1016/j.carbon.2014.10.080

[S10] X. M. Yin, H. J. Li, L. Y. Han, J. C. Meng, J. H. Lu et al., Lightweight and flexible 3D graphene microtubes membrane for high-efficiency electromagnetic-interference shielding. Chem. Eng. J. **387**, 124025 (2020). https://doi.org/10.1016/j.cej.2020.124025

[S11] Y. J. Wan, X. Y. Wang, X. M. Li, S. Y. Liao, Z. Q. Lin et al., Ultrathin densified carbon nanotube film with "metal-like" conductivity, superior mechanical strength, and ultrahigh electromagnetic interference shielding effectiveness. ACS Nano **14**(10), 14134-14145 (2020). https://doi.org/10.1021/acsnano.0c06971

[S12] P. Hu, J. Lyu, C. Fu, W. B. Gong, J. Liao et al., Multifunctional aramid nanofiber/carbon nanotube hybrid aerogel films. ACS Nano **14**(1), 688-697 (2020). https://doi.org/10.1021/acsnano.9b07459

[S13] Q. Song, F. Ye, X. Yin, W. Li, H. Li et al., Carbon nanotube-multilayered graphene edge plane core-shell hybrid foams for ultrahigh-performance electromagnetic-interference shielding. Adv. Mater. **29**(31), 1701583 (2017). https://doi.org/10.1002/adma.201701583

[S14] D. W. Lu, Z. C. Mo, B. H. Liang, L. L. Yang, Z. F. He et al., Flexible, lightweight carbon nanotube sponges and composites for high-performance electromagnetic interference shielding. Carbon **133**, 457-463 (2018). https://doi.org/10.1016/j.carbon.2018.03.061

[S15] F. Shahzad, M. Alhabeb, C. B. Hatter, B. Anasori, S. Man Hong et al., Electromagnetic interference shielding with 2D transition metal carbides (MXenes). Science **353**(6304), 1137-1140 (2016). https://doi.org/10.1126/science.aag2421

[S16] J. Wei, Z. Lin, Z. Lei, Y. Xu, Z. Zhao et al., Lightweight and highly compressible expandable polymer microsphere/silver nanowire composites for wideband electromagnetic interference shielding. ACS Appl. Mater. Interfaces **14**(4), 5940-5950 (2022). https://doi.org/10.1021/acsami.1c20593

[S17] J. Liu, H. B. Zhang, R. Sun, Y. Liu, Z. Liu et al., Hydrophobic, flexible, and lightweight MXene foams for high-performance electromagnetic-interference shielding. Adv. Mater. **29**(38), 1702367 (2017). https://doi.org/10.1002/adma.201702367

[S18] S. Wan, X. Li, Y. Chen, N. Liu, Y. Du et al., High-strength scalable mxene films through bridging-induced densification. Science **374**(6563), 96-99 (2021).

https://doi.org/10.1126/science.abg2026

[S19] Z. Barani, F. Kargar, Y. Ghafouri, S. Ghosh, K. Godziszewski et al., Electrically insulating flexible films with quasi-1D van der waals fillers as efficient electromagnetic shields in the ghz and sub-thz frequency bands. Adv. Mater. **33**(11), e2007286 (2021). https://doi.org/10.1002/adma.202007286
